# Guides or guidelines for interacting and playing with medical complex children: a qualitative documentary research ^1^

**DOI:** 10.1590/1518-8345.6691.4146

**Published:** 2024-07-05

**Authors:** Jéssica Renata Bastos Depianti, Thaís Guilherme Pereira Pimentel, Fernanda Borges Pessanha, Juliana Rezende Montenegro Medeiros de Moraes, Ivone Evangelista Cabral

**Affiliations:** 1 Universidade Federal do Rio de Janeiro, Escola de Enfermagem Anna Nery, Rio de Janeiro, RJ, Brazil.; 2 Scholarship holder at the Conselho Nacional de Desenvolvimento Científico e Tecnológico (CNPq), Brazil.; 3 Scholarship holder at the Coordenação de Aperfeiçoamento de Pessoal de Nível Superior (CAPES), Brazil.; 4 Universidade do Estado do Rio de Janeiro, Faculdade de Enfermagem, Rio de Janeiro, RJ, Brazil.

**Keywords:** Child, Play and Playthings, Social Interaction, Material Resources in Health, Guidelines as Topic, Child Health

## Abstract

**Objectives:**

to identify content on play and interaction with children with special health care needs recommended in clinical guidelines; analyze play and interaction activities applicable to children with special health care needs and complex care requirements.

**Method:**

qualitative documentary research based on guides, protocols, or guidelines on playing and interacting with children with special and living with complex care. Search terms in English (guidelines, playing OR play, complex needs, OR chronic disease) and in Portuguese ( *guia, brincar ou brincadeiras, condições crônicas* ) on the first ten pages of_Google Search ^®^ . Thematic analysis was applied to the information extracted from the documents.

**Results:**

a total of nine documents with similar content were grouped into units of analysis, keeping only the interacting and playing activities applicable to children with special health care needs and living with complex care requirements, namely stimulation of potential, stimulation of adult-child interaction, and stimulation of the senses (touch, sight, and hearing), to be carried out by health professionals and family caregivers in the different care contexts.

**Conclusion:**

interaction and play are potential promoters of adult-child interaction, with application in the stimulating and life-delivering complex care for children.

## Highlights

**(1)** The potential of children with special health care needs.

**(2)** The adult as the object of play and interaction with the child with multiple disabilities.

**(3)** Interacting and playing promote sensory (touch, sight, hearing) and emotional stimulation.

**(4)** The need to overcome the invisibility of children with medical complexity.

**(5)** Knowledge translated into a stimulating and life-delivering Care Guide.

## Introduction

 The knowledge produced from scientific research has consolidated Nursing as a science, with its specialties related to human groups. Pediatric nursing is one of these specialties that is booming in generating evidence to support nursing assistance practices with the child population ^(^^1^^)^ . However, there is a need to translate this evidence, be it scientific, from professional experience, and from user preferences, to improve the health of children and their families in the places where they live and in the different care contexts ^(^^2^^-^^3^^)^ . 

 Knowledge translation is understood from the Canadian perspective ^(^^4^^)^ and defined by the Ministry of Health ^(^^5^^)^ as: “a dynamic and interactive process involving the synthesis, dissemination, exchange, and application of ethics to promote health, strengthening and effectiveness of health services”. To this end, it involves the social responsibility of the researcher and the commitment to transform the results of their research into health actions for the population ^(^^3^^)^ . 

 It is noteworthy that, traditionally, the dissemination of knowledge has favored formats inaccessible to most users or consumers of health services, being more restricted to the scientific community through the publication of articles and the presentation of studies at events. In the search for an efficient practice, reducing the gap between science and assistance is recommended. Among other things, the knowledge-to-action (KTA) model of the knowledge translation approach favors the development of a tool based on the scientific literature pertinent to the content and format. At KTA, the process of producing any tool begins with identifying the need for it through a comprehensive bibliographic search ^(^^6^^)^ . 

 In this search process, the need to have as a starting point the tracking of guiding tools for interaction in care that dynamize playing and play is integrated into caring for Children with Special Health Care Needs living with Complex Care (CSHCN-CC) requirements. In pediatric nursing, tools can mediate content on caring for CSHCN by applying the KTA, but not for those with medical complex care requirements. For example, the Almanac for colostomy reversal ^(^^7^^)^ and the Portuguese version of the short film named “ *Nossas Histórias* ” reveal the diagnosis of the human immunodeficiency virus/Syndrome of Immunodeficiency (HIV/AIDS) acquired by the child ^(^^8^^)^ . In both productions, evidence is transformed into visual languages, respectively static and moving, to mediate implementation strategies. However, their production was based on field research and not on documents as the primary source of information tracked down in the search strategy. 

 Knowledge translation, as one of the implementation science approaches, is widely disseminated globally and begins with searching for specific products for the intended target audience, with content and applicability depending on the local context ^(^^9^^)^ . In Canada, researchers have created an *e-book* for family members of children suffering from chronic pain ^(^^10^^)^ , and others have developed clinical guidelines on the care of children with spinal cord injuries ^(^^11^^)^ . In both cases, knowledge translation proved to be a practical approach for developing CSHCN with chronic illnesses, but none related to playing and playing with children with medical complexity. The latter belong to the subset of children with special healthcare needs and correspond to those with complex chronic conditions and multiple disabilities that determine medical complex care requirements ^(^^12^^)^ . They have total or partial limitations, life-sustaining body technology, and maintain permanent links with medium-sized health services and highly complex daily clinical care. Language, motor, and cognitive barriers can be interpreted as limiting their ability to play with other children and adults. However, it is necessary to access the available interaction channels to maintain their development, reduce anxiety, and promote comfort and well-being based on their potential rather than their limits ^(^^13^^)^ . 

 Regarding tools to guide play and interaction, globally, there are different guides or guidelines disseminated by non-governmental and governmental organizations ^(^^14^^-^^15^^)^ , containing evidence that meets the needs of typical children and little information aimed at the CSHCN audience. In particular, there is a more significant shortage of tools to guide play and interaction for CSHCN-CC, which depends on care ^(^^16^^)^ . When applied, these tools can stimulate psychosocial and affective development, well-being, comfort, and connection with the adult world. In this sense, this study aims to identify content on playing and interacting with CSHCN recommended in clinical guidelines and to analyze play and interaction activities applicable to children with special health care needs living with complex care requirements. 

## Method

### Study design

 Documentary research was carried out in the following stages: choosing the topic, drawing up the work plan, identifying and locating documents, compiling and filing data, analyzing and interpreting the data, and final drafting ^(^^17^^)^ . Documentary research, known in international literature as *documentary* or *document analysis* , is a form of primary research with a qualitative approach, which interrogates and investigates documents through systematic procedures rather than directly with the person. Like any other qualitative research method, document analysis requires examining and interpreting data to elicit meanings, understand new phenomena and develop empirical knowledge. The following documents can be used in this systematic analysis process: advertising leaflets, diaries, manuals, books and brochures, newspaper articles, etc ^(^^17^^)^ . 

### Data collection

 The Google Search ^®^ Platform was chosen as the source for obtaining the documents to operationalise the documentary research procedures. After successive information retrieval tests, the search strategy was applied on September 29, 2022. This platform is an open and extensive repository that allows the retrieval of various materials and sources of information. This search engine is a potential resource for developing research in different areas and geographical regions ^(^^18^^)^ . 

The documents were searched jointly by two researchers with expertise in CSHCN-CC and interacting and playing.

### Inclusion and exclusion criteria

 As for the eligibility of the documents, the inclusion criteria were materials (guides, protocols, or guidelines) that dealt with playing and interacting with children with special health care without delimiting the time frame. Materials to support the development of special education in the school environment, duplicate texts, scientific articles, books, leaflets, and websites were excluded *.*

 The search strategy combined English terms ( *guidelines, playing* or *play* , *complex needs* , OR *chronic disease* ) with the Boolean operator AND *.* The same procedure was implemented with the words in Portuguese ( *guia, brincar* ou *brincadeiras, condições crônicas* ). In both cases, the *PDF* extension was used to refine the search and retrieve only the full-text materials available. 

**Figure 1:**
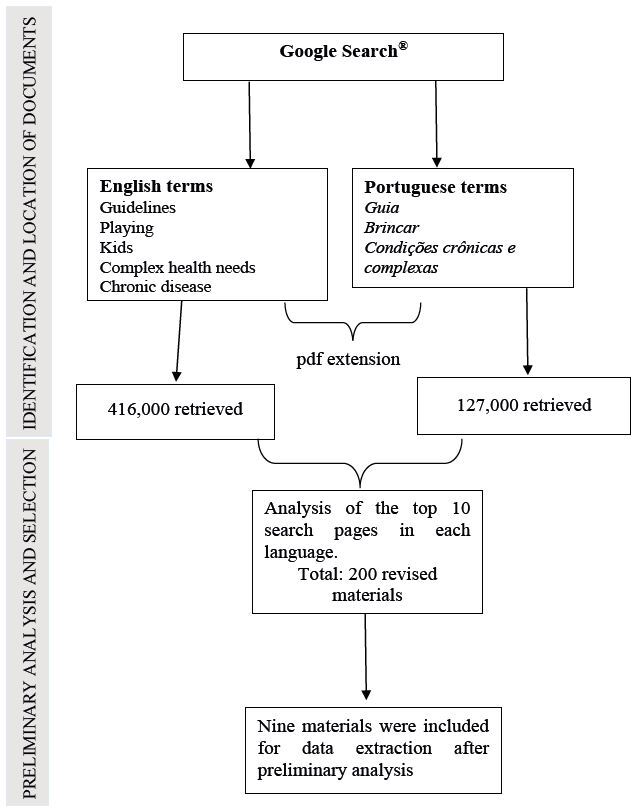
- Flowchart for the location, identification, and preliminary analysis of documents. Brazil, September 2022

 For selecting the materials, the strategy was to analyze the first ten pages of Google Search ^®^ because the search engine›s algorithm shows the first results with the most relevant content. This choice was based on the experience of a previous study in which the results matched most of the words in the search strategy ^(^^16^^)^ . The flowchart below describes the stages of locating, identifying, and selecting documents ( Figure 1 ). 

### Processing and analyzing data

 Extracting the content (compiling and filing the data) was part of the first stage of thematic analysis, corresponding to pre-analysis when the full text was preliminarily read to identify the text’s context, authors, key concepts, nature, logic and internal coherence. At this stage, only the information related to the object of interest ^(^^19^^)^ answered the analytical question: What are the play and interaction activities recommended in guides for children with special health care? 

 Next, the contents with similar meanings were grouped into units of analysis in each text retrieved, keeping only the contents applicable to CSHCN-CC, as shown in Figure 2 . 

**Figure 2:** - Procedure for analyzing the documents retrieved on playing-interacting with CSHCN-CCC 2010-2020. Brazil, September 2022

**Title/Country/Year**	**Extracted content**	**Recommended play-interact activities**	**Potential application to CSHCN-CC** ^*^ **- Children with special health care with medical complex care requirements**
Guidelines and Strategies for Children with Special Needs/United States/ 2010	It presents guidelines so that CSHCNs have a welcoming environment to play and develop their skills and the empowerment of families. In addition, it illustrates how actions and play should be implemented for each condition (chronic, sensory, or cognitive).	To create a welcoming environment for playing and illustrating play by adapting it so that children with different conditions (chronic, sensory, or cognitive) can participate.	Bring a toy closer to the child. Develop actions that stimulate the senses (touch, sight, and hearing). Talk to the child about what is being done with them. Repeat the same play more than once.
Care for Child Development. Guide for Clinical Practice/Global Recommendations/2012	*Clinical sessions:* Play and communicate with the child using activities and toys appropriate to their age and condition (not specific); be sensitive to the child›s conditions and respond appropriately.	Ask the caregiver questions to understand how he plays and communicates with the child at home and observe their interaction.	Be sensitive to the child’s condition and respond appropriately to their signals.	Take advantage of the child’s experience by calling him by name or showing him an object of interest, looking him in the eye. Observe the responses of children and caregivers to recreational and communication activities.	Using a colored object (cup) in play and social coexistence makes the child more alert and active. Call the child by name. Talk to them until they respond with sounds or gestures.
*O Direito de Brincar. Um Guia Prático para Criar Oportunidades Lúdicas e Efetivar o Direito de Brincar* /Brazil/2013	The material highlights inclusion and equality so that all children have the right to play. It reinforces that play facilitators must ensure that children with disabilities are included in art, music, dance, theatre, and stories.	All children should have their right to play guaranteed and be included in art, music, and stories.	Adults are needed to be agents of playing in groups of children.	Play and sing music, move and dance for the child. Offer objects that the child can grasp and that make sounds (rattles). Tell stories using interactive books. Exploring natural environments and contact with plants, insects and pets. Manage programs and play spaces near the bed. Focus on potential rather than lack of ability.
*Incluir Brincando. Guia do Brincar Inclusivo* /Brazil/2012	Selection of toys, plays, and games that allow children to interact and participate, regardless of their characteristics.	Select toys, plays, and games that facilitate the child’s interaction and participation.	Toys must have bright colors, as they are stimulating and help those with low vision perceive contrasts and those that produce a sound: storybooks, music, and puppets.	Select toys, plays, and games that facilitate the child’s interaction and participation. Selecting toys with bright colors is stimulating for children with low vision to perceive contrasts. Select toys that make a sound. Use stories published in the form of sound and music books Use puppets in storytelling.
*Diretrizes de estimulação precoce: crianças de 0 a 3 anos com atraso no desenvolvimento neuropsicomotor* /Brazil/2016	It lists interactive plays and actions for children aged 0 to 3 to stimulate the neuromotor development of children with microcephaly.	Stimulate residual vision, tactile and sound perception during play with other children and caregivers, presenting objects within a visual field of 20 cm, accompanied by verbal stimulation.	Encourage parents or guardians to interact with the child and establish communication and mutual affection.	Use large toys and objects adapted to the child and their sensory functions (auditory, tactile, proprioceptive, and vestibular) so that they look and move their head (if they have trunk control) towards the object, talking to them Use objects of varying sizes and types, coloured with high contrast (yellow and black and or red and white), bright and illuminated. Use sound toys (rattles) in their field of vision and or horizontally, moving them back and forth slowly. Use different textures for tactile stimulation, for example, grains, sponges, cotton, among others. Provide mobiles, rattles, musical toys to bite, and toys that float. Play hide and seek.
Sit Less, Move More, Sleep Well: Active play guidelines for under-fives/New Zealand/2017	Examples of play with children over five that are important and contribute to the development process are given. The guide includes an approach to children with disabilities without specifying how to do and what to use in play with this group.	Using music, moving colored objects around so the child can look for them, using a bathtub to promote water activities, and encouraging play outside or in parks.	Use music as a sound stimulus. Move colored objects around so that the child looks for them. Put some water in the bathtub so that the child keeps skin contact with the water. Promote water activities in safe conditions. Encourage play outside or in parks whenever there are opportunities.
*Guia ilustrado para cuidadores de crianças com deficiências neuromotoras* /Brazil/2017	It includes information on the management of care for children with neuromotor disabilities. Presents plays and activities, recommending correct positioning that promotes neuromotor development.	Bathing is an excellent opportunity for adult-child interaction. Let the child explore the water and have fun with it. Use sponges with different textures and shapes to apply to the child’s body; use toys or simple objects for bathing. Sieves, cups, and jars can be used for playing and stimulating the child’s perception.	If possible, carry the child looking straight ahead so that they receive a greater variety of stimuli and interact better with the environment.	Squares and parks are excellent for children to meet other children and receive different stimuli from those they already have at home. Like any other child, they will have fun touching plants and animals.	In the bath: Let the child explore the water and have fun with it. Apply sponges with different textures and shapes to the body. Use toys or simple objects during the bath (cups and pots stimulate the child’s perception). In mobility: Carry the child looking straight ahead so that they receive the greatest variety of stimuli and interact with the environment, whenever possible. Encourage them to meet other children outdoors so that they receive different stimuli. Encouraging them to touch plants and animals helps with fun and entertainment.
South Australian Government. Inclusive play: Guidelines for accessible playspaces/Australia/2019	The Guide directs the creation of spaces that promote inclusion and playing, creating sensory and interactive experiences.	Sensory play using objects with different textures.	Parks with inclusive toys, with elements within the child’s reach, and with wheelchair access.	Use objects with different textures in play. Parks with inclusive toys (wheelchair swings) that are within children’s reach.
Sensory Design Guidelines: Inclusive Children’s Treatment Centres/Canada/2020	The material proposes guidelines for designing sensory and inclusive environments in children’s treatment centres to improve experiences and well-being. In addition, it also proposes the development of a sensory environment matrix that will address the five senses (hearing, sight, touch, smell, and taste).	Create outdoor spaces having contact with elements of nature (earth, leaves, water), which contain objects with smooth, rough surfaces and or soft textures; and use music and musical objects to promote interaction with children or ambient music to keep them relaxed, can promote interest, engagement and increase tactile stimulation.	Create outdoor spaces for sensory stimulation using the elements of nature (earth, leaves, water) and surfaces and objects with different textures. Use music and musical objects to promote interaction with the children or ambient music to keep them relaxed.

Paper extracte* :CSHCN-CC = Children with special health care living with complex care

In a new analytical move, the units were regrouped and indexed into three themes: sensory stimulation, stimulation of adult-child interaction, and stimulation of potentialities.

## Results

Of the 200 materials that resulted from the search strategy, nine met the eligibility criteria and answered the analytical question. Four of the nine documents were produced in Brazil and address guidelines that ensure the right to play. Inclusive play proposes activities to stimulate the neuromotor development of children with microcephaly and delayed neuromotor development, in general. Out of nine, two were published in North American countries (the United States and Canada) and recommend, respectively, guidelines for children with multiple disabilities and inclusive sensory guidelines for children in treatment centers. Two guides published in Australia and New Zealand recommend, respectively, inclusive play and active play for children under five. The United Nations Children’s Fund (UNICEF) clinical guide recommends a set of guidelines to stimulate the development of children with special health care.

 Activities with the potential to promote potentialities, interaction, and sensory stimulation (touch, hearing, and sight) for CSHCN-CC were grouped into three themes, as shown in Figure 3: stimulation of potentialities, with one subtheme (society’s belief in the child’s potential for coexistence); stimulation of adult-child interaction with two subthemes (voice and body expression of the adult as mediators of play-interaction; management of play); and sensory stimulation (touch, sight and hearing), with four subthemes (Purposes of toys and objects, positioning of toys and objects, play-interaction in bathing, stimulating mobility, whenever possible). 

**Figure 3:** - Recommended play and interaction activities for children with special health care living with complex care requirements. Brazil, September 2022

*Stimulating potencialities*
*Society’s belief in the child’s potential for coexistence* Encouragement to play outside the home, in parks or other public areas, whenever possible. Exposure to inclusive toys (wheelchair swings) that are within their reach. Encouragement to play in the playroom or outside the hospital environment with other children. Keeping the focus on potentialities rather than lack of ability. Promoting the potential to play with water activities in safe conditions.
*Stimulating adult-child interaction*
Adult voice and body expression as mediators of play-interaction The voice is an object of play used to stimulate recognition of the person talking to the child; it informs about what is being done and facilitates interaction. Talking until a response is obtained (sound or gestures). Calling the child by his/her name. Repeating the same play more than once. Moving your body dancing for the child. Playing hide and seek.
*Stimulating adult-child interaction*
Play management Manage programs and play spaces near the bed. Use toys, plays, and games that facilitate the child’s interaction and participation.
*Sensory stimulation (touch, hearing and sight)*
Purposes of toys and objects: That produces sound with solid colors to stimulate the perception of contrasts among those with low vision. Large and adapted to the sensory functions (hearing, tactile, proprioceptive, and vestibular) to stimulate the gaze and movement of the head (if there is trunk control) towards the object: with varied sizes and types, colored with high contrast (yellow and black and or red and white), bright and illuminated; with different textures to promote tactile stimulation, for example, grains, sponges, cotton, among others; that is colorful (cups), used in the play to stimulate interaction and make the child more alert and active; using music (to play and sing in the environment) as a sound resource for interaction and relaxation; for touching and floating, such as mobiles, rattles, and musical objects. Sound and musical books for use in stories. Interactive books and puppets as resources for storytelling and getting the child’s attention. In outdoor spaces, contact with nature (plants, earth, leaves, water) and contact with insects and pets promote sensorial stimulation. Positioning toys and objects: Closer (to touch, hearing, and sight) to the child. Moving them around so that the child looks for them. Within the visual field and or horizontally, moving sound toys (rattles) slowly back and forth. Within the hand’s reach so the child can grasp (hold) and produce sounds (rattles). Play-interaction in the bath: To explore water and have fun with it. Use sponges with different textures and shapes on the body. Maintain skin-to-skin contact with the water by adding a little water to the bathtub. Use simple toys or objects (cups and pots) that stimulate the child’s perception. Encouraging mobility whenever possible Transport the child, keeping them facing forward so that they receive the greatest variety of stimuli and interact with the environment. Promote encounters with other children in external environments so that they receive different stimuli.

## Discussion

 Most of the time, the focus of care is on meeting the survival needs of the CSHCN-CC, prioritizing nursing assistance that guarantees unobstructed breathing, safe feeding, intact skin, and medication administration for continuous use, among other aspects. This survival care is continuous, prolonged and intense, drawing nursing professionals and family caregivers to it. In this sense, this section discusses the findings based on the assumptions of Collière’s Care ^(^^20^^)^ and the concept of play according to Walter Benjamin ^(^^21^^)^ . 

 Collière ^(^^20^^)^ understands care as life-giving and invites life, describing it in seven types without ranking them by the degree of relevance. The first, *maintenance* , relates to everyday life’s psychological, emotional, and social needs. The second, *restorative* , aims to recover from the illness through treatment. The third, *comfort* , refers to the renewal and integration of the experience that promotes well-being. The fourth, *emancipation* , contributes to enhancing the person’s self-image to strengthen their sense of identity and belonging to the social group in which they live. *Compensation* , the fifth type of care, involves actions to replace what has not yet been acquired and compensation for what the person cannot do for themselves. *Pacification* , the sixth type, aims to rest and release tensions, making it possible to calm down what is causing turbulence and unrest. The seventh type, *stimulation* , aims to stimulate the fundamental capacities of the senses, perception, and representation; it highlights a variety of forms of care capable of producing expectations, desires, interests, and affection. Play, in Collière’s understanding, is maintenance care ^(^^20^^)^ . However, according to the unique characteristics of the CSHCN-CC, play is a substantive activity that mobilizes vitality and the potential for interaction; therefore, more than one type of care applies from Collière’s perspective. 

Sometimes, these children’s limited motor and psychosocial conditions prevent adults, nursing professionals, or family caregivers from realizing the potential they have to participate in interactions and play that promote well-being and connection with the world. The limiting and medical complex condition constitutes barriers to playing and interacting, which is not recognized as a valuable activity in the community, at school, or in health services, especially in hospitals. In this sense, it is necessary to look at the uniqueness of playing/play as a human activity that connects the CSHCN-CC with the world of adults.

 To better understand the place of play in children’s lives, Benjamin’s thinking ^(^^21^^)^ is the benchmark. According to the author, play promotes liberation, creating a small world for the child that is their own and is surrounded by the world of adults. It is from this moment of creation that pleasure is extracted, and it is this that makes the child feel free. Play is the mimetic faculty whose essence is the innovation of “doing it again” and repeating the experience that play provides. It involves the child’s ability to step out of themselves, lose themselves in the other (this other may be a situation, place, person, object, or word), and return to themselves in a way transformed by the experience of being another. In other words, when they play, they can dilute themselves in space, place, and time to give meaning to the object they manipulate or the countless roles it can perform. 

 In this way, interactions are limited by the low responsiveness to stimuli normally expected of typical children ^(^^22^^-^^23^^)^ . 

 There is a tendency in health services to value the biomedical approach, prioritizing the organic issues inherent in the child’s condition. In Canada, institutional programs prioritize “being a child”, seeking the child’s well-being, rest, and comfort, centered on the psychosocial care model. For this reason, they invest in the child’s potential for interactional development in their social world ^(^^13^^,^^24^^)^ . 

 It should be noted that, for CSHCN-CC, care should stimulate their potential. However, there is an ontological belief about the potential of children with cerebral palsy to interact during play. A study highlighted that some professionals do not believe in the child’s potential and express their ontological view by saying there is no need to take the child out of bed to play, as the child was doing nothing ^(^^24^^)^ . A bedridden child with a severe neurological deficit; even though he had toys on his bed, no one interacted with him, gave him attention or even played with him. The child struggled to reach one of the toys, got tired, and went back to look at them without any adult using the objects as a resource to promote interaction mediated by play ^(^^24^^)^ . Both situations reinforce the need to break down preconceptions so that adults engage in activities that stimulate the potential of CSHCN-CC. This evidence must be translated into interaction tools in life-giving care so that the objects of playing and play can produce meaning for the child. 

It should be noted that these activities require a safe environment, whether they take place in water or any other environment that poses a risk to the child’s physical integrity. It is also necessary to encourage health professionals to interact with the child and family members so that they do not leave these children confined to their homes. Taking them to environments outside the home, such as parks, for example, helps to provide stimuli mediated by animals and plants and the opportunity to use inclusive toys (wheelchair swings) that are within their reach. However, the playroom and environments outside the hospital are also recommended during prolonged hospitalisation. When translated into a care tool, this body of evidence favours the development of the interactional potential of the CSHCN-CC with the adult, be it a nursing professional or family caregiver, who is part of their social world in the hospital environment.

 For Collière, care implies a personal and social commitment from the caregiver to the person cared for ^(^^20^^)^ . Caring may only be a simple or trivial gesture, but it is an act of life. In addition, it represents a variety of activities aimed at sustaining and or maintaining life, that is, enabling people to continue living to their full potential ^(^^25^^)^ . In this sense, creating spaces that stimulate the potential of the CSHCN-CC is an act of life and investment in their potential and not in their lack of ability ^(^^13^^)^ . 

When it comes to stimulating potential, society needs to be welcoming to the CSHCN-CC, and people need to believe in the potential for coexistence that they provide, encouraging them to play outside the home (park or other public areas), in the hospital playroom or areas outside the hospital. These environments provide opportunities to meet others, as well as the possibility for them to interact with other children and toys. In addition, when there is a safe condition, activities should be carried out in the water when incorporated as evidence in care tools.

 The stimulating potential of the CSHCN-CC, mediated by play, represents the regulation of play, one of the fundamental rights provided for in the Brazilian Child and Adolescent Statute (CAS) (Law No. 8,069/1990)(26), to make one of the facets of care effective. In addition, it complies with other legal guidelines set out in the Statute for People with Disabilities (Law No. 13,146/2015) ^(^^27^^)^ and the obligation to install playrooms in pediatric hospitalization units (Law No. 11,104/2005)(28). In addition to this, the Brazilian National Policy of Comprehensive Child Healthcare ( *Política Nacional de Atenção Integral à Saúde à Criança* , PNAISC) emphasizes that it is the responsibility of the family, the community, society in general, and the Public Authorities to ensure that children’s rights are fulfilled, such as the right to life, leisure, family and community life, participation and respect for freedom ^(^^29^^)^ . The legal evidence, corresponding to regulations in the form of laws and government ordinances, underpins CSHCN-CC’s right to play and needs to be translated into care-guiding tools. 

 For the CSHCN-CC group, which depends on others to play and interact, there is a need to encourage initiatives that *stimulate adult-child interaction* . Thus, activities mediated by the adult’s voice and bodily expression become the object of play, how interaction is established, and a dialogue captured by the emission of signals ^(^^30^^-^^31^^)^ . It is recommended that, during care, the CSHCN-CC be called by their caregiver (professional or family member) by their name, that he talks to him about what will be done, play hide-and-seek, and repeat these actions until responses are obtained, such as the emission of guttural sounds or spontaneous or reflex movement. 

 By repeating the same game more than once, such as hide-and-seek or moving their bodies (like a dance), a small world of their own is created, surrounded by the world of adults, which provides pleasure and promotes liberation. The *stimulation of adult-child interaction* , when repeated, according to Benjamin, means *mimesis* ; that is, there is a recording of similarities and not equality. “Doing it again” involves the child’s ability to step out of themselves, lose themselves in the other (this other may be a situation, a place, a person, an object or a word) and return to themselves in a way transformed by the experience of being another. Similar experiences allow the child to become familiar with the external world, with ideas, feelings and actions whose potential can be transformative, allowing them to glimpse new possibilities for relationships between worlds ^(^^31^^)^ . 

Providing similar experiences for CSHCN-CC, especially those hospitalized for long periods, increases the relational repertoire that keeps them connected to the world and expands their social interaction. Therefore, managing play programs and spaces, as well as toys and games near the bed, which facilitate interaction and participation of the child according to their ability, can be enriching and provide consistent and different sensory stimuli.

 When it stimulates the senses (touch, sight, and hearing), it is essential to know the purpose of each toy, especially those objects that apply to CSHCN-CC. Therefore, it must be borne in mind that these materials must have stimulating, bright, illuminated, and contrasting colours, especially for those with low vision. In addition, sound objects of varying sizes (large balls) and different textures stimulate the movement of the head and or eyes towards the object, making the child more alert and active in the interaction, as well as being adapted to the sensory functions that are to be stimulated ^(^^13^^)^ . In addition to these toys, activities that stimulate the senses include singing or playing children’s songs, storytelling, reading to the child, and watching TV ^(^^30^^)^ . When interacting with CSHCN-CC, play needs to be accompanied by sensory stimuli (hearing, sight and touch) to elicit pleasurable responses that can be seen in facial expressions (smiling, looking) or other parts of the body. The stimulus awakens interest in sound, touch, and other elements in the environment, leading the child to seek them out, directing their gaze to the source of the voice and touch, for example ^(^^31^^-^^32^^)^ . 

 The human voice is a powerful object of play, whether in conversation, storytelling or (sound) musical books, just as music (playing, singing in the environment) is a sound resource for interaction and relaxation. Another way of stimulating the senses is to use elements of nature (earth, leaves, water) in outdoor spaces so that children do not live in the confinement imposed by clinical complexity. The data aligns with a study carried out with children with cerebral palsy who took part in a multisensory storytelling group in which they had significant participation and interaction ^(^^32^^-^^33^^)^ . 

However, to promote sensory stimulation, play objects must be made available horizontally within the child’s sound and visual field, and adults move sound toys (such as the rattle) slowly so that the child looks for them. Objects that stimulate grasping should be kept within the child’s hands.

 The sensations experienced with sensory stimulation (touch, hearing, and sight) are triggered by the neurosensory nerve receptor when it becomes aware of, recognizes, and interprets this stimulus. In other words, this set of sensory experiences, whether through music, colourful and sonorous toys/objects, and different textures, allows children to perceive and increase their level of awareness regarding communication and pleasurable emotions ^(^^12^^-^^13^^)^ . 

 From Collière’s perspective, stimulating care aims to awaken the most fundamental capacities, which are feeling, hearing, and seeing, to develop the senses and motor skills. Stimulating care creates expectations, desires, interests, and affective reactions, the starting point for constructing thought ^(^^20^^)^ . 

 Consequently, arousing pleasurable responses in CSHCN-CC is strategic stimulation care when the caregiver (nursing staff and family) creates a welcoming, pleasurable, and well-being-promoting environment, which contributes to creating affective bonds based on the experiences of these sensations ^(^^12^^,^^30^^)^ . For CSHCN-CC, care must be transmitted in an interaction with the other through touch and body-to-body contact, the human voice, allowing them to find their place in the world and feel safe. For example, bathing (in the bathtub or bed) creates the opportunity to stimulate the senses, as it allows them to explore the water and have fun when they touch it, and using objects (sponges) and toys with different textures that attract attention. 

 However, before implementing them, it is necessary to know the child’s clinical condition and the possibility of doing so. Regarding mobility, if the child can be transferred from the bed to a wheelchair or carried on the lap, they should be positioned to look forward and thus receive more stimuli and interactions from external environments and people they are not used to ^(^^13^^)^ . When experienced playfully, all these practices arouse pleasure and satisfaction and are exchanged in the reciprocity of mutual enrichment when interacting and playing ^(^^13^^)^ . Therefore, interaction and playing are life-giving care, that is, care that invites life, restoring meaning to the children who receive it, especially for the CSHCN-CC, who are still invisible to society regarding their potential. 

 To this end, it is necessary to rethink the model of care linked exclusively to the biomedical paradigm to introduce new practices based on an emerging paradigm of care centered on the needs of hospitalized children ^(^^34^^)^ . Therefore, health institutions and health professionals must take a closer look at scientific production on play and interaction in the mediation of care. The development of interactional activities in care can promote sociability, awaken the potential of CSHCN-CC, reduce the gap between assistance and science, and foster efficient and humanized practice. To this end, care guides are tools with content that can instrumentalize professionals and family members in stimulating activities. 

## Conclusion

The clinical guides retrieved in the search strategy showed two groups of content. First, many deal with play and interaction, emphasizing the activities for children with special health care (CSHCN). Most of them have microcephaly, delayed neuromotor development, low vision, and multiple disabilities, among others). The second is indicative of activities that have the potential to be applied to CSHCN living with complex care (CSHCN-CC) requirements. This evidence, originating from documents as primary sources of analysis, reveals the potential for translation and transferability to produce an integrative tool for interacting in the daily care of CSHCN-CC.

Playing and interacting with CSHCN-CC can be grouped into three activities in this tool. The first are those that stimulate the child’s potential based on society’s and people’s ontological belief in the ability to live together in the social space. The second is based on the need for adult-child interaction as a source of stimulation with the adult’s voice and body expression, and in the management of play, the latter brings together activities that promote sensory stimulation according to the types and the purposes of toys and objects; as well as the care when positioning the child, during bathing, and mobility.

It is also important to emphasize the need for health professionals and family members to break with the invisibility of these children, investing in and valuing their achievements. The specific content for stimulating the senses (touch, sight, hearing) of CSHCN-CC can be mediated by objects of play and games as long as the knowledge is translated into a guiding tool for health professionals (nursing, in particular) and family caregivers, to promote stimulating and life-giving care.

 As a limitation of the study, the guides and guidelines found do not address the racial, gender, and socioeconomic issues of children with special health care. In this sense, there is a need for research that can cover ethnic-racial diversity, gender, and social equity, broadening the spectrum of social inclusion in the construction of knowledge about CSHCN-CC. In addition, the search was limited to English and Portuguese, with the search strategy applied to a single source of information, the Google Search ^®^ *.*
