# Supplementary material for: Guides or guidelines for interacting and playing with medical complex children: a qualitative documentary research
Source: Rev Lat Am Enfermagem. 2024 Jul 5;32:e4146. doi: 10.1590/1518-8345.6691.4146 (PMC11251688; doi:10.1590/1518-8345.6691.4146)
Supplement: Translation

# Translation: Guías o directrices para interactuar y jugar con niños con condiciones clínicas complejas: investigación cualitativa documental ^1^

**DOI:** 10.1590/1518-8345.6691.4145

**Authors:** Jéssica Renata Bastos Depianti, Thaís Guilherme Pereira Pimentel, Fernanda Borges Pessanha, Juliana Rezende Montenegro Medeiros de Moraes, Ivone Evangelista Cabral

**Affiliations:** 1 Universidade Federal do Rio de Janeiro, Escola de Enfermagem Anna Nery, Rio de Janeiro, RJ, Brasil.; 2 Becaria del Conselho Nacional de Desenvolvimento Científico e Tecnológico (CNPq), Brasil.; 3 Becaria de la Coordenação de Aperfeiçoamento de Pessoal de Nível Superior (CAPES), Brasil.; 4 Universidade do Estado do Rio de Janeiro, Faculdade de Enfermagem, Rio de Janeiro, RJ, Brasil.

**Keywords:** Niño, Juego e Implementos de Juego, Interacción Social, Recursos Materiales en Salud, Guías como Asunto, Salud Infantil

## Abstract

**Objetivos:**

identificar contenido sobre juego e interacción con niños con necesidades de especiales atención en salud recomendados en guías clínicas; analizar las actividades de juego e interacción que se pueden implementar niños con necesidades especiales de atención en salud y demandas de cuidados clínicamente complejos.

**Método:**

investigación cualitativa documental basada en guías, protocolos o directrices para jugar e interactuar con niños con necesidades especiales de atención en salud. Búsqueda de los términos en inglés ( *guidelines, playing* o *play, complex needs* OR *chronic disease* ) y en portugués ( *guia, brincar* o *brincadeiras, condições crônicas* ), en las primeras 10 páginas de *Google Search* ^®^ . Se aplicó análisis temático a la información extraída de los documentos.

**Resultados:**

se agruparon en unidades de análisis nueve documentos con contenido similar, se extrajeron solo las actividades para interactuar y jugar que se pueden implementar con niños con necesidades especiales de atención en salud y demandas de cuidados clínicamente complejos, a saber: estimular las potencialidades, estimular la interacción adulto-niño y estimular los sentidos (tacto, visión y oído), que realizan los profesionales de la salud y los cuidadores familiares en diferentes contextos de cuidado.

**Conclusión:**

interactuar y jugar pueden promover la interacción adulto-niño e implementarse en el cuidado estimulante y vivificante de niños con condiciones clínicas complejas.

## Destacados

**(1)** Potencialidades del niño con necesidades especiales de atención en salud clínicamente complejas.

**(2)** El adulto como objeto de juego e interacción con el niño con discapacidades múltiples.

**(3)** Interactuar y jugar promueven la estimulación sensorial (tacto, visión, oído) y afectiva.

**(4)** Es necesario superar la invisibilidad de los niños con condiciones clínicas complejas.

**(5)** Conocimiento traducido en una Guía de Cuidados estimulante y vivificante.

## Introducción

 El conocimiento producido a partir de la investigación científica ha consolidado a la Enfermería como ciencia, que cuenta con diferentes especialidades relacionadas con los grupos humanos. La enfermería pediátrica es una de las especialidades que produce cada vez más evidencia que sirve de base para la atención de enfermería de la población infantil ^(^^1^^)^ . Sin embargo, es necesario traducir esa evidencia, ya sea científica, producto de la experiencia profesional y de las preferencias de los usuarios, para mejorar la salud de los niños y sus familias en el lugar donde viven y en diferentes contextos de atención ^(^^2^^-^^3^^)^ . 

 La traducción del conocimiento se considera, desde la perspectiva canadiense ^(^^4^^)^ , y es definida por el Ministerio de Salud ^(^^5^^)^ como: “un proceso dinámico e interactivo, que implica la síntesis, la difusión, el intercambio y la aplicación de la ética para promover la salud, el fortalecimiento y la eficacia de los servicios de salud”. Eso requiere la responsabilidad social del investigador y el compromiso para transformar los resultados derivados de su investigación en acciones de salud para la población ^(^^3^^)^ . 

 Cabe destacar que, tradicionalmente, la difusión del conocimiento privilegia formatos que no son accesibles para la mayoría de los usuarios de los servicios de salud, se restringen más a la comunidad científica, se realiza a través de la publicación de artículos y la presentación de trabajos en eventos. Por lo tanto, para lograr una práctica eficiente, se recomienda reducir la brecha entre ciencia y atención. Entre otros, el modelo del conocimiento para la acción (MCA) del enfoque de traducción del conocimiento privilegia el desarrollo de una herramienta basada en la literatura científica relevante sobre contenido y formato. En el MCA, el proceso de producción de cualquier herramienta comienza por identificar la necesidad de elaborarla, realizando una búsqueda bibliográfica exhaustiva ^(^^6^^)^ . 

 En este proceso de búsqueda, sostenemos que es necesario tener como punto de partida el seguimiento de herramientas que orienten la interacción en el cuidado, que incentiven el juego y el juego integrado al proceso de cuidado de Niños con Necesidades Especiales de Atención en Salud con demandas de cuidados clinicamente complejos (NNEAS-CCC). En enfermería pediátrica hay herramientas disponibles que median contenidos para la atención de NNEAS aplicando el MCA, pero no para aquellos con demandas de atención clínicamente complejas. Por ejemplo, el Almanaque para la reversión de la colostomía ^(^^7^^)^ y el cortometraje “Nuestras Historias” para revelar el diagnóstico del virus de la inmunodeficiencia humana/Síndrome de Inmunodeficiencia (VIH/SIDA) adquirido por el niño ^(^^8^^)^ . En ambas producciones, se observa que se transformó la evidencia en lenguaje visual estáticos y en movimiento, respectivamente, para mediar estrategias de implementación. Sin embargo, la producción se basó en investigaciones de campo y no en documentos, como fuente primaria de información consultada en una estrategia de búsqueda. 

 La traducción del conocimiento, como uno de los enfoques de la ciencia de implementación, está ampliamente difundida en el contexto global y comienza con la búsqueda de productos específicos para el público objetivo previsto, con contenido y aplicabilidad, según contexto local ^(^^9^^)^ . En Canadá, hay investigadores que crearon un libro electrónico para familiares de niños que sufren de dolor crónico ^(^^10^^)^ , y otros que desarrollaron directrices clínicas sobre el cuidado de niños con lesiones de la médula espinal ^(^^11^^)^ . En ambos casos, la traducción del conocimiento demostró ser un enfoque eficaz para el desarrollo de NNEAS con enfermedades crónicas, pero ninguno se relaciona con el juego y el juego con NNEAS-CCC. 

 Los NNEAS-CCC pertenecen al subconjunto de niños con necesidades especiales de atención en salud, y corresponden a aquellos con condiciones crónicas complejas y/o discapacidades múltiples que implican demandas de atención clínicamente complejas ^(^^12^^)^ . Tienen limitaciones totales o parciales, utilizan tecnología corporal para seguir con vida, mantienen un vínculo permanente con los principales servicios de salud y reciben atención clínica diaria de alta complejidad. Se puede interpretar que las barreras lingüísticas, motoras y cognitivas los limitan a la hora de jugar con otros niños y adultos. Sin embargo, es necesario acceder a los canales de interacción disponibles para mantener el curso de su desarrollo, reducir la ansiedad y promover el confort y el bienestar, basándose más en sus potencialidades que en sus limitaciones ^(^^13^^)^ . 

 En cuanto a las herramientas que orientan el juego y la interacción, a nivel mundial, hay diferentes guías o directrices difundidas por organizaciones no gubernamentales y gubernamentales ^(^^14^^-^^15^^)^ , que contienen evidencia para satisfacer las necesidades del niño promedio y poca información dirigida al público NNEAS. En particular, hay una mayor escasez de herramientas para orientar el juego y la interacción con los NNEAS-CCC, que dependen completamente de los cuidados ^(^^16^^)^ . Esas herramientas, cuando se usan, tienen el potencial de promover la estimulación del desarrollo psicosocial y afectivo, el bienestar, la comodidad y la conexión con el mundo adulto. Por lo tanto, el presente estudio tiene como objetivo identificar contenido sobre juego e interacción con NNEAS recomendados en guías clínicas; analizar actividades de juego e interacción que se puedan implementar con niños con necesidades especiales de atención en salud y demandas de atención clínicamente complejas. 

## Método

### Diseño de estudio

 Se realizó una investigación documental, cuyas etapas fueron: elección del tema, elaboración del plan de trabajo, identificación y localización de documentos, recolección y registro de datos, análisis e interpretación de datos y redacción final ^(^^17^^)^ . La investigación documental, conocida en la literatura internacional como *documentary* o *document analysis* , es una forma de investigación primaria, con enfoque cualitativo, que consulta y estudia documentos en lugar de consultar directamente a la persona, mediante procedimientos sistemáticos. Como cualquier otro método de investigación cualitativa, el análisis de documentos requiere que se examinen e interpreten los datos para obtener significados, comprender nuevos fenómenos y desarrollar conocimiento empírico. En ese proceso de análisis sistemático se pueden utilizar los siguientes documentos: folletos publicitarios, agendas, manuales, libros, artículos periodísticos, etc. ^(^^17^^)^ . 

### Recolección de datos

 Para operacionalizar los procedimientos de búsqueda de documentos, se eligió como fuente la plataforma *Google Search* ^®^ . La estrategia de búsqueda se aplicó el 29 de septiembre de 2022, luego de realizar sucesivas pruebas de recuperación de información. Esta plataforma es un repositorio abierto y amplio que permite la recuperación de una variedad de materiales y fuentes de información. Este motor de búsqueda presenta un recurso potencial para el desarrollo de investigaciones en diferentes áreas y regiones geográficas ^(^^18^^)^ . 

La búsqueda de documentos la realizaron de forma conjunta dos investigadores con experiencia en el tema NNEAS-CCC y en la interacción y el juego.

### Los criterios de inclusión y exclusión

 En cuanto a la elegibilidad de los documentos, los criterios de inclusión fueron materiales (guías, protocolos o directrices) que abordaran el juego y la interacción con niños con necesidades especiales de atención en salud, sin límite temporal. Se excluyeron materiales para el apoyo del desarrollo de la educación especial en el ámbito escolar, textos duplicados, artículos científicos, libros, folletos y sitios *web* . 

 En la estrategia de búsqueda se aplicó una combinación de términos en inglés ( *guidelines, Playing* o *Play, Complex Needs* OR *Chronic Disease* ) combinados por el operador booleano AND. Se implementó el mismo procedimiento con las palabras en portugués ( *guia, brincar* o *brincadeiras, condições crônicas* ). En ambos casos, se eligió la extensión *pdf* para afinar la búsqueda y recuperar sólo material disponible en texto completo. 

 Para seleccionar el material, se adoptó como estrategia revisar las primeras diez páginas de *Google Search* ^®^ , debido a que el algoritmo de ese buscador presenta como primeros resultados los que tienen el contenido más relevante. Esta opción se basó en la experiencia de un estudio anterior cuyos resultados coincidieron con la mayoría de las palabras de la estrategia de búsqueda ^(^^16^^)^ . El siguiente diagrama de flujo describe los pasos para localizar, identificar y seleccionar documentos ( Figura 1 ). 

**Figura 1:** - Diagrama de flujo de localización, identificación y análisis preliminar de documentos. Brasil, septiembre 2022

### Procesamiento y análisis de datos

 La extracción del contenido (recopilación y registro de datos) formó parte de la primera etapa del análisis temático, correspondiente al preanálisis, cuando se realizó la lectura preliminar del texto completo para identificar el contexto; autores; conceptos clave; naturaleza, lógica y coherencia interna de los textos. En esta etapa solo se extrajo información relacionada al objeto de interés ^(^^19^^)^ y que respondía a la pregunta analítica: ¿cuáles son las actividades de juego e interacción recomendadas en las guías para niños con necesidades especiales de atención en salud? 

 Luego, el contenido con significado similar se agrupó en unidades de análisis en cada texto recuperado, se extrajo solo con el contenido aplicable a NNEAS-CCC, como se muestra en la Figura 2 . 

**Figura 2:** - Procedimiento de análisis de documentos recuperados sobre juego-interacción con NNEAS-CCC 2010-2020. Brasil, septiembre de 2022

**Título/País/Año**	**Contenido extraído**	**Recomendación de actividades de juego-interacción**	**Potencial de aplicación para NNEAS-CCC** ^*^ **- Niños con necesidades especiales de atención en salud con demandas de cuidados clínicamente complejos**
*Guidelines and Strategies for Children with Special Needs* /Estados Unidos / 2010	Brinda orientación para que los cuenten con un ambiente acogedor para jugar, desarrollar sus habilidades y empoderar a sus familias. Además, explica, para cada condición (crónica, sensorial o cognitiva), cómo se deben implementar acciones y juegos.	Ambiente acogedor para el juego y explica juegos, adaptándolos para que puedan participar niños con diferentes condiciones (crónicas, sensoriales o cognitivas).	Colocar un juguete más cerca del niño; Desarrollar acciones que estimulen los sentidos (tacto, visión y oído); Hablar con el niño sobre lo que se hace con él; Repetir el mismo juego más de una vez.
*Care for Child Development. Guide for Clinical Practice* /Recomendación mundial/2012	Sesiones clínicas: jugar y comunicarse con el niño utilizando actividades y juguetes adecuados para su edad y condición (no especifica); ser sensible a las condiciones del niño y responder adecuadamente a ellas.	Hacerle preguntas al cuidador para comprender cómo juega y se comunica con el niño en casa, y observar sus interacciones.	Ser sensible a las condiciones del niño, respondiendo adecuadamente a sus señales.	Aprovechar la experiencia del niño llamándolo por su nombre o mostrándole algún objeto que le interese, mirándolo a los ojos. Observar las respuestas de los niños y los cuidadores al juego y las actividades de comunicación.	Usar un objeto colorido (vaso) en el juego y en la interacción social hace que el niño esté más alerta y activo; Llamar al niño por su nombre; Hablar con él hasta obtener una respuesta mediante sonidos o gestos.
*O Direito de Brincar. Um Guia Prático para Criar Oportunidades Lúdicas e Efetivar o Direito de Brincar* /Brasil/2013	El material destaca la inclusión y la igualdad, para que todos los niños tengan derecho a jugar. Recalca que los facilitadores del juego deben asegurarse de que los niños con discapacidad participen en el arte, la música, la danza, el teatro y las historias.	Hay que garantizar el derecho de todos los niños al juego y que participen en el arte, la música y las historias.	Es necesario que los adultos sean agentes de juego en los grupos de niños.	Tocar y cantar canciones, moverse y bailar para el niño; Ofrecerle objetos que el niño pueda agarrar (prensión) y que emitan sonidos (sonajeros); Contarle historias usando libros interactivos; Explorar ambientes naturales y tener contacto con plantas, insectos, mascotas; Gestionar programas y espacios de juego cerca de la cama; Enfocarse en las potencialidades y no en la falta de habilidad.
*Incluir Brincando. Guia do Brincar Inclusivo* /Brasil/2012	Elección de juguetes y juegos que les permitan a los niños interactuar y participar, independientemente de sus características.	Elegir juguetes, y juegos que faciliten la interacción y participación del niño.	Los juguetes deben tener colores vivos, dado que son estimulantes y ayudan a los niños con poca visión a percibir contrastes, además deben producir algún sonido; libros de historias, música y títeres.	Elegir juguetes y juegos que faciliten la interacción y participación del niño; Elegir juguetes con colores brillantes es estimulante, les permite a los niños con poca visión percibir los contrastes; Elegir juguetes que produzcan algún sonido; Utilizar historias publicadas en formato de libros sonoros y con música; Utilizar títeres al contar historias.
*Diretrizes de estimulação precoce: crianças de 0 a 3 anos com atraso no desenvolvimento neuropsicomotor* /Brasil/2016	Enumera juegos y acciones interactivas para niños de 0 a 3 años, con el objetivo de estimular el desarrollo neuromotor de los niños con microcefalia.	Estimular la visión residual, la percepción táctil y sonora al jugar con otros niños y cuidadores, presentando objetos dentro de un campo visual de 20 cm, acompañados de estimulación verbal.	Potenciar el aporte de los padres o tutores, para que interactúen con el niño y haya comunicación y afecto mutuo;	Utilizar juguetes y objetos grandes adaptados al niño, a sus funciones sensoriales (auditivas, táctiles, propioceptivas y vestibulares), para que mire y mueva la cabeza (si tiene control del tronco) hacia el objeto, mientras conversa con él Utilizar objetos de diferentes tamaños y tipos, con colores de alto contraste (amarillo y negro y/o rojo y blanco), brillantes y luminosos; Utilizar juguetes sonoros (sonajeros) en su campo visual y/u horizontalmente, moviéndolos hacia adelante y hacia atrás lentamente; Utilizar diferentes texturas para la estimulación táctil, por ejemplo: semillas, esponjas, algodón, etc.; Proporcionar móviles, sonajeros, juguetes musicales para morder y juguetes suspendidos; Jugar a las escondidas
*Sit Less, Move More, Sleep Well: Active play guidelines for under-fives* / Nueva Zelanda /2017	Señalan ejemplos de juegos con niños mayores de cinco años que son importantes y contribuyen al proceso de desarrollo. La guía incluye a los niños con discapacidad, pero no especifica cómo hacer ni qué utilizar para jugar con este grupo.	Usar música; mover objetos coloridos para que el niño pueda buscarlos; utilizar una bañera para promover actividades en el agua; fomentar el juego fuera de casa o en los parques.	Utilizar la música como recurso estimulador sonoro; Mover objetos de colores para que el niño los busque; Poner un poco de agua en la bañera para que la piel del niño entre en contacto con el agua; Promover actividades en el agua en condiciones seguras; Fomentar el juego fuera de casa o en los parques siempre que sea posible.
*Guia ilustrado para cuidadores de crianças com deficiências neuromotoras* /Brasil/2017	Información sobre el cuidado y manejo de niños con discapacidad neuromotora. Presenta juegos y actividades, y recomienda posiciones correctas que favorecen el desarrollo neuromotor.	El baño es una excelente oportunidad para la interacción entre adultos y niños. Dejar que el niño explore el agua y se divierta con ella. Utilizar esponjas con diferentes texturas y formas para pasarlas por el cuerpo del niño; usar juguetes u objetos sencillos para el baño. Se pueden utilizar coladores, vasitos y frasquitos para jugar y estimular la percepción del niño.	Si es posible, sostener al niño mirando hacia adelante para que reciba una mayor variedad de estímulos e interactúe mejor con el entorno.	Las plazas y los parques son excelentes para que los niños conozcan a otros niños y reciban estímulos diferentes a los que ya tienen en casa. Como cualquier otro niño, se divertirá tocando plantas y animales.	Durante el baño: Dejar que el niño explore el agua y se divierta con ella; Pasarle por el cuerpo esponjas con diferentes texturas y formas; Utilizar juguetes u objetos sencillos durante el baño (los vasitos y frasquitos estimulan la percepción del niño). En movimiento: Llevar al niño mirando hacia adelante para que reciba la mayor variedad de estímulos e interactúe con el entorno, siempre que sea posible; Promover encuentros con otros niños, en ambientes externos para que reciban diferentes estímulos; Animarlo a tocar plantas y animales contribuye a que se divierta y entretenga.
*South Australian Government. Inclusive play: Guidelines for accessible playspaces* /Australia/2019	La Guía orienta la creación de espacios que promuevan la inclusión y el juego, que creen experiencias sensoriales e interactivas.	Juegos sensoriales utilizando objetos con diferentes texturas.	Parques con juguetes inclusivos, con elementos al alcance del niño y con acceso para silla de ruedas.	Usar objetos con diferentes texturas en los juegos; Parques con juguetes inclusivos (columpios con sillas de ruedas) y que estén al alcance de los niños.
*Sensory Design Guidelines: Inclusive Children’s Treatment Centres* /Canadá/2020	El material propone pautas para diseñar ambientes sensoriales e inclusivos en centros de cuidado infantil para mejorar las experiencias y el bienestar. Además, propone el desarrollo de un modelo de ambiente sensorial que aborde los cinco sentidos (oído, visión, tacto, olfato y gusto).	Crear espacios al aire libre para que tengan contacto con elementos de la naturaleza (tierra, hojas, agua) y que cuenten con objetos, superficies lisas, rugosas y/o texturas suaves; utilizar música y objetos musicales para promover la interacción con los niños o música ambiental para mantenerlos relajados puede promover el interés, la participación y aumentar la estimulación táctil.	Crear espacios al aire libre para estimular los sentidos a través de elementos naturales (tierra, hojas, agua) y superficies y objetos de diferentes texturas; Utilizar música y objetos musicales para promover la interacción con los niños o música ambiental para mantenerlos relajados.

* NNEAS-CCC = Niño con necesidades especiales de atención en salud con demandas de cuidados clínicamente complejos

En un nuevo movimiento analítico, las unidades fueron reagrupadas e indexadas en tres temas: estimular los sentidos, estimular la interacción adulto-niño y estimular las potencialidades.

## Resultados

De los 200 materiales que se obtuvieron como resultado de la estrategia de búsqueda, nueve cumplían con los criterios de elegibilidad y respondían a la pregunta analítica. Cuatro de los nueve documentos fueron producidos en Brasil y abordan directrices que garantizan el derecho a jugar. El juego inclusivo propone actividades para estimular el desarrollo neuromotor de niños con microcefalia y retraso en el desarrollo neuromotor, en general. Dos de los nueve fueron publicados en países de América del Norte (Estados Unidos y Canadá) y recomiendan, respectivamente, directrices para niños con discapacidades múltiples y directrices sensoriales inclusivas para niños atendidos en centros de tratamiento. Dos guías publicadas en Australia y Nueva Zelanda recomiendan el juego inclusivo y el juego activo para niños menores de cinco años, respectivamente. La guía clínica del Fondo de las Naciones Unidas para la Infancia (UNICEF) recomienda un conjunto de directrices para estimular el desarrollo de niños con necesidades especiales de atención en salud.

 Las actividades con potencial para promover las potencialidades, la interacción y la estimulación sensorial (tacto, oído y visión) de NNEAS-CCC se agruparon en tres temas, como se muestra en la Figura 3: estimular las potencialidades, con un subtema (creencia de la sociedad en el potencial de convivencia del niño); estimular la interacción adulto-niño con dos subtemas (la voz y expresión corporal del adulto como mediadores de la interacción-juego; gestión del juego); y estimular los sentidos (tacto, visión y oído), con cuatro subtemas (propósitos de los juguetes y objetos, posición de los juguetes y objetos, interactuar-jugar durante el baño, estimular la movilidad, siempre que sea posible) 

**Figura 3:** - Actividades de juego e interacción recomendadas para niños con necesidades especiales de atención en salud con demandas de cuidados clínicamente complejos. Brasil, septiembre de 2022

*Estimular las potencialidades*
*Creencia de la sociedad en el potencial de convivencia del niño* fomentar el juego fuera del hogar, en parques u otras áreas públicas, siempre que sea posible; exponerlo a juguetes inclusivos (columpios con sillas de ruedas) y que estén a su alcance; estimular el desarrollo de juegos en la ludoteca o fuera del entorno hospitalario con otros niños; mantener el foco en las potencialidades y no en la falta de habilidad; promover el potencial para jugar con actividades en el agua, en condiciones seguras.
*Estimular la interacción adulto-niño*
*La voz y expresión corporal del adulto como mediadores del juego-interacción* la voz es un objeto de juego que se utiliza para estimular el reconocimiento de la persona que habla con el niño; informa lo que se está haciendo y facilita la interacción; hablar hasta obtener una respuesta (sonido o gestos); llamar al niño por su nombre; repetir el mismo juego más de una vez; mover el cuerpo bailando para el niño; jugar a las escondidas. *Gestión del juego* gestionar programas y espacios de juego cerca de la cama; utilizar juguetes y juegos que faciliten la interacción y participación del niño.
*Estimular los sentidos (tacto, oído y visión)*
*Propósitos de los juguetes y objetos:* que produzcan sonido; con colores fuertes, para estimular la percepción de contrastes de los niños con poca visión; grandes y adaptados a las funciones sensoriales (auditiva, táctil, propioceptiva y vestibular), para estimular la mirada y el movimiento de la cabeza (si tiene control del tronco) hacia el objeto; con diferentes tamaños y tipos, con colores de alto contraste (amarillo y negro y/o rojo y blanco), brillantes y luminosos; con diferentes texturas para favorecer la estimulación táctil, por ejemplo: semillas, esponjas, algodón, etc.; que sean coloridos (vaso), utilizarlos en el juego para estimular la interacción, haciendo que el niño esté más alerta y activo; utilizar la música (para tocar y cantar; en el ambiente), como recurso sonoro para interactuar y relajarse; para morder y que esté suspendido, como móviles, sonajeros y objetos musicales; libros sonoros y musicales para contar historias; libros interactivos y títeres, como recursos para contar historias y llamar la atención del niño; en espacios al aire libre, el contacto con la naturaleza (plantas, tierra, hojas, agua), el contacto con insectos y mascotas promueven la estimulación sensorial. *Posición de los juguetes y objetos:* más cerca (del tacto, el oído y la vista) del niño; moverlos para que el niño los busque; dentro del campo visual y/o horizontalmente, mover juguetes sonoros (sonajeros) lentamente, hacia adelante y hacia atrás; al alcance de la mano para que pueda agarrarlo (prensión) y producir sonidos (sonajeros). *Interactuar-jugar durante el baño:* explorar el agua y divertirse con ella; pasarle esponjas con diferentes texturas y formas por el cuerpo; mantener el contacto de la piel con el agua, poner poca agua en la bañera; utilizar juguetes u objetos sencillos (vasitos y frasquitos) que estimulen la percepción del niño. *Estimular la movilidad, siempre que sea posible* transportar al niño manteniéndolo mirando hacia adelante, para que reciba la mayor variedad de estímulos e interactúe con el entorno; promover encuentros con otros niños, en ambientes externos para que reciban diferentes estímulos.

## Discusión

 La mayoría de las veces, los cuidados se centran en atender las necesidades de supervivencia de los NNEAS-CCC, se prioriza el cuidado de enfermería que garantiza una respiración despejada, nutrición segura, piel íntegra y administración de medicamentos de uso continuo, entre otras cosas. Estos cuidados de supervivencia son continuos, prolongados e intensos, y captan el cuidado de los profesionales de enfermería y de los cuidadores familiares. Por ende, en esta sección se discuten los hallazgos basándose en los supuestos del Cuidado de Collière ^(^^20^^)^ y la concepción de juego de Walter Benjamin ^(^^21^^)^ . 

 Collière ^(^^20^^)^ considera que los cuidados son vivificantes e invitan a la vida, y los describe en siete tipos, pero no los clasifica por grado de relevancia. El primero, de *mantenimiento* , está relacionado con las necesidades psicológicas, afectivas y sociales que forman parte de la vida cotidiana. La segunda, de *reparación* , tiene como objetivo la recuperación de la enfermedad mediante el tratamiento. El tercero, el *confort* , se refiere a la renovación e integración de la experiencia que promueve el bienestar. El de *opinión* , corresponde al cuarto, contribuye a la valoración de la imagen que la persona tiene de sí misma para fortalecer el sentido de identidad y pertenencia al grupo social en el que vive. El de *compensación* , el quinto tipo de cuidado, implica acciones sustitutivas de lo que aún no ha sido adquirido y/o compensación de lo que la persona aún no es capaz de hacer por sí misma. El de *apaciguamiento* , el sexto tipo, tiene como objetivo descansar y liberar tensiones, que permiten apaciguar lo que causa perturbación y lo que genera malestar. El séptimo, de *estimulación* , tiene como objetivo estimular las capacidades fundamentales, de los sentidos, la percepción y la representación; se destaca una variedad de cuidados capaces de producir expectativas, deseos, intereses y afectos. Jugar, para Collière, es un cuidado de mantenimiento ^(^^20^^)^ . 

Sin embargo, según las características únicas de los NNEAS-CCC, el juego es una actividad sustantiva que moviliza la vitalidad y el potencial de interacción; por lo tanto, desde la perspectiva de Collière se aplica más de un tipo de cuidado.

En ocasiones, las limitadas condiciones motrices y psicosociales de estos niños impiden que los adultos, profesionales de enfermería o cuidadores familiares perciban las potencialidades que tienen para participar en interacciones y juegos que promueven el bienestar y la conexión con el mundo. La condición limitante y clínicamente compleja constituye una barrera para jugar e interactuar, por ende, tanto en la comunidad, como en la escuela y en los servicios de salud, especialmente en el hospital, no se reconoce lo valiosas que son esas actividades. Por lo tanto, es necesario observar la singularidad del jugar/juego como actividad humana que conecta al NNEAS-CCC con el mundo de los adultos.

 Para comprender mejor el lugar del juego en la vida del niño, tomamos como marco de referencia el pensamiento de Benjamin ^(^^21^^)^ . Según el autor, el juego promueve la liberación, crea para el niño un pequeño mundo propio que está rodeado por el mundo de los adultos. De ese momento de la creación surge el placer y es lo que hace que el niño se sienta libre. El juego es la facultad mimética cuya esencia es la innovación de “hacerlo de nuevo”, de repetir la experiencia que brinda el juego. Implica la capacidad del niño de salir de sí mismo, perderse en el otro (ese otro puede ser una situación, lugar, persona, objeto o palabra) y volver a sí mismo de una manera transformada por la experiencia de ser otro. Es decir, al jugar logra diluirse en el espacio, el lugar y el tiempo para darle sentido al objeto que manipula o a los innumerables roles que este puede desempeñar. 

 Por lo tanto, las interacciones están limitadas por la baja capacidad de respuesta a los estímulos que normalmente se espera de los niños típicos ^(^^22^^-^^23^^)^ . 

 Hay una tendencia en los servicios de salud a valorar el enfoque biomédico, que prioriza las cuestiones orgánicas inherentes a la condición del niño. En Canadá, los programas institucionales priorizan “ser niño”, buscan su bienestar, descanso y comodidad, se enfocan en el modelo de atención psicosocial. Por esta razón, invierten en el potencial de desarrollo de interacción del niño en su mundo social ^(^^13^^,^^24^^)^ . 

 Cabe destacar que, en el caso de los NNEAS-CCC, el cuidado debe estimular sus potencialidades. Sin embargo, existe una creencia ontológica sobre el potencial que tienen los niños con parálisis cerebral para interactuar durante el juego. Un estudio destacó que algunos profesionales no creen en el potencial del niño y expresaron su visión ontológica cuando dijeron que no era necesario sacarlo de la cama para jugar, ya que no hacía nada ^(^^24^^)^ . Por ejemplo, un niño estaba postrado en la cama, con déficit neurológico severo, a pesar de que tenía juguetes en su cama, nadie interactuaba con él, le prestaba cuidado o simplemente jugaba. El niño luchaba por alcanzar uno de los juguetes, se cansaba y volvía a mirarlos, sin que ningún adulto utilizara los objetos como recurso para promover la interacción mediada por el juego ^(^^24^^)^ . Ambas situaciones refuerzan la necesidad de romper con los prejuicios para que los adultos puedan participar en actividades que estimulen las potencialidades de los NNEAS-CCC. Esta evidencia debe traducirse en herramientas de interacción en el cuidado vivificante, de modo que los objetos para jugar y para el juego produzcan significado para el niño. 

Hay que destacar que dichas actividades requieren de un ambiente seguro, ya sea que se realicen en o con agua o en otro ambiente que represente un riesgo para la integridad física del niño. También es necesario incentivar a los profesionales de la salud a interactuar con el niño, al igual que a los familiares, para que no dejen a estos niños confinados en sus hogares. Llevarlos a ambientes fuera de casa, como parques, por ejemplo, ayuda a proporcionar estímulos mediados por animales y plantas, además brinda la oportunidad de utilizar juguetes inclusivos (columpios con sillas de ruedas) que estén a su alcance. Sin embargo, si están hospitalizados, especialmente si es por mucho tiempo, también se recomienda recurrir a la ludoteca y ambientes externos del hospital. Dicho conjunto de evidencias, traducidas en una herramienta de cuidado, favorece el desarrollo del potencial de interacción del NNEAS-CCC con el adulto, ya sea un profesional de enfermería o un familiar cuidador, que forma parte de su mundo social en el ambiente hospitalario.

 Para Collière, el cuidado implica un compromiso personal y social del cuidador hacia la persona cuidada ^(^^20^^)^ . Cuidar puede ser un gesto simple o trivial, pero no deja de ser un acto de vida. Además, representa una variedad de actividades cuyo objetivo es promover y/o preservar la vida, es decir, permitir que la persona siga viviendo con todo su potencial ^(^^25^^)^ . Por ende, crear espacios que estimulen el potencial de los NNEAS-CCC es un acto de vida y de inversión en su potencial y no en su falta de habilidad ^(^^13^^)^ . 

 Para *estimular las potencialidades* , la sociedad debe acoger a los NNEAS-CCC y las personas deben creer en el potencial de convivencia que tienen, animarlos a jugar fuera de casa (parque u otras áreas públicas), en la ludoteca del hospital o en áreas externas del hospital. Estos entornos crean oportunidades para encontrarse con otros, así como también la posibilidad de que los niños interactúen con otros niños y juguetes. Además, cuando las condiciones sean seguras, hay que realizar actividades en el agua, cuando se incorporen como evidencia en las herramientas de cuidado. 

*Estimular las potencialidades* del NNEAS-CCC, a través del juego, representa la regulación del juego, uno de los derechos fundamentales previstos en el Estatuto del Niño y del Adolescente en Brasil (ENA) (Ley n.º 8.069/1990) ^(^^26^^)^ , para implementar una de las facetas del cuidado. Además, cumple con otras directrices legales previstas en el Estatuto de las Personas con Discapacidad en Brasil (Ley n.º 13.146/2015) ^(^^27^^)^ y la obligación de instalar ludotecas en las unidades de hospitalización pediátrica (Ley n.º 11.104/2005) ^(^^28^^)^ . Por otro lado, la Política Nacional de Atención Integral a la Salud del Niño en Brasil (PNAISN) destaca que la familia, la comunidad, la sociedad en general y el Poder Público son los responsables de garantizar que se cumplan los derechos del niño, tales como a la vida, al ocio, a la convivencia familiar y a la comunidad, a la participación y al respeto a la libertad ^(^^29^^)^ . La evidencia legal, correspondiente a regulaciones en forma de ley y disposiciones gubernamentales, respalda el derecho de los NNEAS-CCC a jugar y debe traducirse en herramientas orientadoras para la atención. 

 Para los NNEAS-CCC, que dependen totalmente de otros para jugar e interactuar, es necesario fomentar iniciativas que *estimulen la interacción adulto-niño* . Por ende, las actividades mediadas por la voz y la expresión corporal del adulto se convierten en objeto de juego, medio por el cual se establece una interacción y un diálogo captado por la emisión de señales ^(^^30^^-^^31^^)^ . Se recomienda que, durante la prestación del cuidado, el cuidador (profesional o familiar) del NNEAS-CCC lo llame por su nombre, que hable con él y le informe lo que hará, juegue a las escondidas y repita esas acciones hasta obtener respuestas, como la emisión de sonidos guturales o movimientos espontáneos o reflejos. 

 Al repetir más de una vez el mismo juego, como las escondidas o mover el cuerpo (a modo de baile), se crea para ellos un pequeño mundo propio rodeado del mundo de los adultos, que les proporciona placer y promueve la liberación. La *estimulación de la interacción adulto-niño* , cuando se produce de forma repetida, según Benjamin significa *mímesis* , es decir, se produce el registro de similitudes y no de la igualdad. “Hacerlo de nuevo” implica la capacidad del niño de salir de sí mismo, perderse en el otro (este otro puede ser una situación, un lugar, una persona, un objeto o una palabra) y volver a sí mismo de una manera transformada por la experiencia de ser otro. Experiencias similares permiten que el niño se familiarice con el mundo externo, con ideas, sentimientos y acciones, cuyo potencial puede ser transformador, permitiéndole vislumbrar nuevas posibilidades de relación entre los mundos ^(^^31^^)^ . 

Brindarles experiencias similares a los NNEAS-CCC, especialmente a los que tienen hospitalizaciones prolongadas, aumenta el repertorio relacional que los mantiene conectados con el mundo y amplía su convivencia social. Por ende, la gestión de programas y espacios de juego, así como de juguetes y juegos cercanos a la cama, que faciliten la interacción y participación del niño en función de sus habilidades, puede resultar enriquecedora, además de brindarle estímulos sensoriales consistentes y diferentes.

 Para *estimular los sentidos (tacto, visión y oído)* , es importante conocer los propósitos de cada juguete, especialmente aquellos objetos que se usan con los NNEAS-CCC. Por lo tanto, es necesario tener en cuenta que estos materiales deben tener colores estimulantes, brillantes, luminosos y contrastantes, especialmente para los niños con poca visión. Además, los objetos sonoros de distintos tamaños (pelotas grandes) y diferentes texturas estimulan el movimiento de la cabeza y/o los ojos hacia el objeto, haciendo que el niño esté más alerta y activo en la interacción, al igual que los que se adaptan a las funciones sensoriales que se pretende estimular ^(^^13^^)^ . Además de esos juguetes, se recomiendan otras actividades para estimulan los sentidos, como cantar o reproducir canciones infantiles, contar historias y leerle al niño, mirar televisión ^(^^30^^)^ . El juego en la interacción con el NNEAS-CCC debe ir acompañado de estímulos sensoriales (oído, visión y tacto) para provocar respuestas de placer que se puedan observar en la expresión facial (sonrisa, mirada) u otras partes del cuerpo. El estímulo despierta interés por el sonido, el tacto y otros elementos del ambiente, y lleva al niño a buscarlos, dirigiendo su mirada al origen de la voz y del contacto, por ejemplo ^(^^31^^-^^32^^)^ . 

 La voz humana es un poderoso objeto de juego, ya sea en la conversación, la narración de historias y los libros musicales (sonoros), al igual que la música (tocar, cantar en el ambiente) representa un recurso sonoro para la interacción y la relajación. Otras formas de estimular los sentidos es utilizar elementos de la naturaleza (tierra, hojas, agua) en espacios exteriores para que el niño no viva en el confinamiento impuesto por la complejidad clínica. Los datos coinciden con los de un estudio realizado con niños con parálisis cerebral que participaron en un grupo de narración multisensorial de historias, en el que se observó una importante participación e interacción de los mismos ^(^^32^^-^^33^^)^ . 

Sin embargo, para promover la estimulación sensorial, es esencial poner los objetos de juego horizontalmente dentro del campo visual y sonoro del niño; y que los adultos muevan lentamente los juguetes sonoros (como sonajeros) para que el niño pueda buscarlos. Los objetos que favorezcan el agarre deben mantenerse al alcance de las manos del niño.

 Las sensaciones experimentadas con la estimulación sensorial (tacto, oído y visión) son desencadenadas por el receptor del nervio neurosensorial al tomar conciencia, reconocer e interpretar este estímulo. En otras palabras, este conjunto de experiencias sensoriales, ya sea a través de la música, los juguetes/objetos coloridos, sonoros y de diferentes texturas, les permite a los niños percibir que captan y aumentar su nivel de conciencia sobre la comunicación y las emociones de placer ^(^^12^^-^^13^^)^ . 

 Desde la perspectiva de Collière, los cuidados de estimulación tienen como objetivo despertar las capacidades más fundamentales, que son sentir, oír y ver, para desarrollar los sentidos y la capacidad motora. El cuidado estimulante genera expectativas, deseos, interés y reacciones afectivas, que son el punto de partida para la construcción del pensamiento ^(^^20^^)^ . 

 Por ende, despertar respuestas de placer en los NNEAS es un cuidado de estimulación estratégico cuando el cuidador (equipo de enfermería y/o familia) crea un ambiente acogedor, placentero, que promueve el bienestar, lo que contribuye a la creación de vínculos afectivos basados en las experiencias de esas sensaciones ^(^^12^^,^^30^^)^ . Para el NNEAS-CCC el cuidado debe transmitirse en la interacción con el otro a través del tacto y el contacto cuerpo a cuerpo, de la voz humana, que les permiten encontrar su lugar en el mundo y sentirse seguros. Por ejemplo, el baño (en bañera o cama) crea la oportunidad de estimular los sentidos, dado que les permite a los niños explorar el agua para divertirse cuando la tocan; y utilizar objetos (esponja) y juguetes con diferentes texturas que les llamen la atención. 

 Sin embargo, antes de implementar esas actividades es necesario conocer el estado clínico del niño y si se puede implementar de esta forma. En cuanto a la movilidad, si el niño puede ser trasladado de la cama a la silla de ruedas o llevado en el regazo, hay que colocarlo de manera que el niño mire hacia adelante, para que reciba más estímulos e interacciones de ambientes externos y personas a las que no está acostubrado ^(^^13^^)^ . 

 Todas estas prácticas, cuando se llevan a cabo de forma lúdica, generan placer, satisfacción y se transforman en la reciprocidad del enriquecimiento mutuo al interactuar y jugar ^(^^13^^)^ . Por lo tanto, la interacción y el juego son cuidados vivificantes, es decir, invitan a la vida, devolviendo un significado a los niños que lo reciben, especialmente para los NNEAS-CCC, dado que para la sociedad aún son invisibles en lo que respecta a su potencial. 

 Por lo tanto, es necesario repensar el modelo de cuidado vinculado exclusivamente al paradigma biomédico, para introducir nuevas prácticas basadas en un paradigma emergente del cuidado centrado en las necesidades del niño hospitalizado ^(^^34^^)^ . Para ello, es necesario que tanto las instituciones de salud como los profesionales de la salud recurran a las producciones científicas sobre el juego y la interacción en la mediación del cuidado. El desarrollo de actividades de interacción en el cuidado puede promover la sociabilidad, despertar el potencial de los NNEAS-CCC, reducir la brecha entre el cuidado y la ciencia y fomentar una práctica eficiente y humanizada. Por lo tanto, las guías de cuidados constituyen herramientas cuyos contenidos pueden ayudar a los profesionales y las familias a realizar actividades estimulantes. 

## Conclusión

Las guías clínicas obtenidas a partir de la estrategia de búsqueda indicaron que hay dos grupos de contenidos. El primero trata sobre el juego y la interacción destacando las actividades que hay que realizar con los niños con necesidades especiales de atención en salud (NNEAS) (microcefalia, retraso en el desarrollo neuromotor, poca visión, discapacidades múltiples). El segundo, indica las actividades con el potencial como para ser implementadas con NNEAS con demandas de cuidados clínicamente complejos (NNEAS-CCC). Esta evidencia, proveniente de documentos como fuentes primarias de análisis, revela el potencial de traducción y transferibilidad para la producción de una herramienta que integre la interacción en el cuidado diario de NNEAS-CCC.

En esta herramienta, el juego y la interacción con NNEAS-CCC se puede agrupar en tres conjuntos de actividades. En el primero, están las que estimulan las potencialidades del niño basadas en la creencia ontológica de la sociedad y de las personas en la capacidad de convivir en el espacio social. El segundo, se basa en la necesidad de interacción adulto-niño como fuente de estimulación con la voz y expresión corporal del adulto y la gestión del juego. El último reúne actividades que promueven la estimulación sensorial, según el tipo y la finalidad de los juguetes y objetos; el cuidado de la posición que debe tener el niño, durante el baño y en el traslado.

También es importante destacar que es necesario que los profesionales de la salud y los familiares acaben con la invisibilidad de estos niños, invirtiendo y valorando sus logros. Los contenidos específicos para estimular los sentidos (tacto, visión, oído) de NNEAS-CCC pueden estar mediados por objetos lúdicos y juegos, siempre y cuando el conocimiento se traduzca en una herramienta orientadora para los profesionales de la salud (enfermeros, en particular) y familiares cuidadores, para promover el cuidado estimulante y vivificante.

 Se considera una limitación del estudio que las guías y directrices encontradas no aborden las cuestiones raciales, de género y socioeconómicas de los niños con necesidades especiales de atención en salud. Por lo tanto, es necesario que se realicen investigaciones que puedan abarcar la diversidad étnico-racial, la equidad de género y social, y amplíen el espectro de la inclusión social en la construcción de conocimiento sobre NNEAS-CCC. Además, la búsqueda se llevó a cabo solo en inglés y portugués, y la estrategia de búsqueda se realizó en una única fuente de información, *Google Search* ^®^ *.*

# Translation: Guias ou diretrizes para interagir e brincar com crianças clinicamente complexas: uma pesquisa qualitativa documental ^1^

**DOI:** 10.1590/1518-8345.6691.4147

**Authors:** Jéssica Renata Bastos Depianti, Thaís Guilherme Pereira Pimentel, Fernanda Borges Pessanha, Juliana Rezende Montenegro Medeiros de Moraes, Ivone Evangelista Cabral

**Affiliations:** 1 Universidade Federal do Rio de Janeiro, Escola de Enfermagem Anna Nery, Rio de Janeiro, RJ, Brasil.; 2 Bolsista do Conselho Nacional de Desenvolvimento Científico e Tecnológico (CNPq), Brasil.; 3 Bolsista da Coordenação de Aperfeiçoamento de Pessoal de Nível Superior (CAPES), Brasil.; 4 Universidade do Estado do Rio de Janeiro, Faculdade de Enfermagem, Rio de Janeiro, RJ, Brasil.

**Keywords:** Criança, Jogos e Brinquedos, Interação Social, Recursos Materiais em Saúde, Guias como Assunto, Saúde da Criança

## Abstract

**Objetivos:**

identificar conteúdos sobre brincar e interagir com crianças com necessidades de saúde especiais recomendados em guias clínicos; analisar as atividades de brincar e interagir aplicáveis às crianças com necessidades de saúde especiais e demandas de cuidados clinicamente complexas.

**Método:**

pesquisa qualitativa documental baseada em guias, protocolos ou diretrizes sobre brincar e interagir com crianças com necessidades de saúde especiais. Busca dos termos em inglês ( *guidelines, playing ou play, complex needs* OR *chronic disease* ) e em português (guia, brincar ou brincadeiras, condições crônicas), nas 10 primeiras páginas do *Google Search* ^®^ . Aplicou-se a análise temática às informações extraídas dos documentos.

**Resultados:**

agruparam-se nove documentos com conteúdo similares em unidades de análise, mantendo-se somente as atividades do interagir e brincar aplicáveis às crianças com necessidades de saúde especiais e demandas de cuidados clinicamente complexas, a saber: estimulação das potencialidades, estimulação da interação adulto-criança e estimulação dos sentidos (tato, visão e audição), a serem realizadas por profissionais de saúde e familiares cuidadores nos diferentes contextos de cuidado.

**Conclusão:**

o interagir e o brincar são potenciais promotores da interação adulto-criança, com aplicação no cuidado estimulador e vivificante de crianças clinicamente complexas.

## Destaques

**(1)** Potencialidades da criança com necessidades de saúde especiais clinicamente complexas.

**(2)** O adulto como objeto de brincar e interagir com a criança com deficiências múltiplas.

**(3)** Interagir e brincar promovem estimulação sensorial (tato, visão, audição) e afetiva.

**(4)** Necessidade de superar a invisibilidade de crianças clinicamente complexas.

**(5)** Conhecimento traduzido em uma Guia de Cuidado estimulador e vivificante.

## Introdução

 O conhecimento produzido a partir de pesquisas científicas tem consolidado a Enfermagem como ciência, com suas diferentes especialidades relacionadas aos grupos humanos. A enfermagem pediátrica é uma dessas especialidades em franca expansão na geração de evidências para sustentar a prática assistencial da enfermagem com a população infantil ^(^^1^^)^ . Porém, há necessidade de se proceder à tradução dessas evidências, sejam elas científicas, da experiência profissional e das preferências dos usuários, para a melhoria da saúde das crianças e suas famílias no lócus onde vivem e nos diferentes contextos de cuidados ^(^^2^^-^^3^^)^ . 

 Por tradução de conhecimento compreende-se, na perspectiva canadense ^(^^4^^)^ e definida pelo Ministério da Saúde ^(^^5^^)^ , como: “um processo dinâmico e interativo, que envolve a síntese, a disseminação, o intercâmbio e a aplicação da ética para promover saúde, fortalecimento e efetividade dos serviços de saúde”. Para tanto, envolve a responsabilidade social do pesquisador e o comprometimento de transformar os resultados advindos de suas pesquisas em ações de saúde para a população ^(^^3^^)^ . 

 Destaca-se que, tradicionalmente, a disseminação de conhecimento tem privilegiado formatos inacessíveis à maioria dos usuários ou consumidores dos serviços de saúde, ficando mais restrito à comunidade científica, por meio da publicação de artigos e da apresentação de trabalhos em eventos. Nesse sentido, na busca de uma prática eficiente, recomenda-se diminuir a lacuna entre a ciência e a assistência. Entre outros, o modelo do conhecimento para ação (MCA) da abordagem de tradução de conhecimento privilegia a elaboração de ferramenta fundamentada na literatura científica pertinente ao conteúdo e formato. No MCA, o processo de produção de qualquer ferramenta inicia com a identificação da necessidade de elaborá-la, numa busca bibliográfica abrangente ^(^^6^^)^ . 

 Nesse processo de busca, defende-se a necessidade de se ter, como ponto de partida, o rastreamento de ferramentas orientadoras da interação no cuidado, que sejam dinamizadoras do brincar e da brincadeira integrada ao processo de cuidar de Crianças com Necessidades de Saúde Especiais, com demandas de cuidados clinicamente complexas (CRIANES-CCC). Na enfermagem pediátrica, estão disponíveis ferramentas mediadoras de conteúdos sobre o cuidar de CRIANES aplicando-se o MCA, mas não para aquelas que possuem demandas de cuidados clinicamente complexas. Como, por exemplo, o Almanaque para reversão de colostomia ^(^^7^^)^ e o curta-metragem “Nossas Histórias” para a revelação do diagnóstico do vírus da imunodeficiência humana/Síndrome da Imunodeficiência (HIV/aids) adquirida pela criança ^(^^8^^)^ . Em ambas as produções se observa a transformação de evidências em linguagens visuais, respectivamente estática e em movimento, para mediar estratégias de implementação. Porém, sua produção baseou-se em pesquisa de campo e não com documentos, como fonte primária de informação rastreada em estratégia de busca. 

 A tradução do conhecimento, como uma das abordagens da ciência de implementação, é amplamente difundida no contexto global, e inicia-se com a busca de produtos específicos para o público-alvo a quem se destina, com conteúdo e aplicabilidade, segundo o contexto local ^(^^9^^)^ . No Canadá, pesquisadores criaram um *e-book* para familiares de crianças que sofrem com dor crônica ^(^^10^^)^ , e outros desenvolveram diretrizes clínicas sobre os cuidados de crianças com lesão de medula espinhal ^(^^11^^)^ . Em ambos os casos, a tradução do conhecimento se mostrou uma abordagem eficaz para o desenvolvimento de CRIANES com doenças crônicas, mas nenhuma relacionada ao brincar e à brincadeira com CRIANES-CCC. 

 As CRIANES-CCC pertencem ao subconjunto de crianças com necessidades de saúde especiais, e correspondem àquelas com condições crônicas complexas e/ou deficiências múltiplas que determinam demandas de cuidados clinicamente complexas ^(^^12^^)^ . Elas apresentam limitação total ou parcial, tecnologia corporal de manutenção da vida, e mantém vínculo permanente com os serviços de saúde de média e cuidados clínicos cotidianos altamente complexos. As barreiras linguísticas, motoras e cognitivas podem ser interpretadas como limitadoras para elas se engajarem no brincar com outras crianças e adultos. Entretanto, é preciso acessar os canais de interação disponíveis, para manter o curso do seu desenvolvimento, reduzir a ansiedade e promover conforto e bem-estar, baseando-se mais em suas potencialidades do que em seus limites ^(^^13^^)^ . 

 No que tange às ferramentas orientadoras do brincar e da interação, globalmente, há diferentes guias ou diretrizes disseminadas por organizações não governamentais e governamentais ^(^^14^^-^^15^^)^ , contendo evidências que atendem às necessidades de crianças típicas e poucas informações direcionadas para o público CRIANES. Evidencia-se, em particular, uma maior escassez de ferramentas orientadoras do brincar e interagir para as CRIANES-CCC, totalmente dependente de cuidados ^(^^16^^)^ . Tais ferramentas, quando aplicadas, têm o potencial de promover a estimulação do desenvolvimento psicossocial e afetivo, o bem-estar, o conforto e sua conexão com o mundo do adulto. Nesse sentido, o presente estudo tem como objetivo identificar conteúdos sobre brincar e o interagir com CRIANES, recomendados em guias clínicos; e analisar as atividades de brincar e interagir, aplicáveis às crianças com necessidades de saúde especiais, bem como as demandas de cuidados clinicamente complexas. 

## Método

### Tipo de estudo

 Implementou-se a pesquisa documental nas seguintes etapas: escolha do tema, elaboração do plano de trabalho, identificação e localização dos documentos, compilação e fichamento de dados, análise e interpretação dos dados e redação final ^(^^17^^)^ . A pesquisa documental, conhecida na literatura internacional como *documentary* ou *document analysis* , é uma forma de pesquisa primária, com abordagem qualitativa, que interroga e investiga documentos, ao invés de fazê-lo diretamente com a pessoa, por meio de procedimentos sistemáticos. Como qualquer outro método de pesquisa qualitativa, a análise dos documentos exige que os dados sejam examinados e interpretados para se elicitar significados, compreender fenômenos novos e desenvolver conhecimento empírico. Podem ser usados nesse processo de análise sistemática os seguintes documentos: folhetos de propagandas, agendas, manuais, livros e brochuras, matérias de jornais, etc ^(^^17^^)^ . 

### Coleta de dados

 Para operacionalizar os procedimentos da pesquisa documental, elegeu-se a Plataforma *Google Search* ^®^ como fonte de obtenção dos documentos. A estratégia de busca foi aplicada em 29 de setembro de 2022, após sucessivas testagens na recuperação de informação. Esta plataforma é um repositório aberto e amplo que permite a recuperação de uma variedade de materiais e fontes de informações. Esse buscador apresenta um potencial recurso para o desenvolvimento de pesquisas em diferentes áreas e regiões geográficas ^(^^18^^)^ . 

A busca dos documentos se deu de forma conjunta, por dois pesquisadores com expertise na temática das CRIANES-CCC e no interagir e brincar.

### Critérios de inclusão e exclusão

 Quanto à elegibilidade dos documentos, determinou-se, como critérios de inclusão, materiais (guias, protocolos ou diretrizes) que abordavam o brincar e interagir com crianças com necessidades de saúde especiais, sem delimitação de recorte temporal. Excluíram-se materiais de apoio ao desenvolvimento da educação especial em ambiente escolar, textos duplicados, artigos científicos, livros, folhetos e *websites.*

 Na estratégia de busca aplicou-se a combinação de termos em inglês ( *guidelines, playing* ou *play, complex needs* OR *chronic disease* ) combinadas pelo operador booleano AND *.* O mesmo procedimento foi implementado com as palavras em português (guia, brincar ou brincadeiras, condições crônicas). Em ambos os casos, adotou-se a extensão *pdf* para refinar a busca e recuperar somente os materiais disponíveis com texto completo. 

 Para a seleção dos materiais, adotou-se a estratégia de revisar as dez primeiras páginas do *Google Search* ^®^ pelo fato do algoritmo desse buscador apresentar os primeiros resultados com conteúdo mais relevantes. Tal opção baseou-se em experiências de estudo anterior cujo resultado combinou a maioria das palavras da estratégia de busca ^(^^16^^)^ . O fluxograma abaixo descreve as etapas de localização, identificação e seleção dos documentos ( Figura 1 ). 

**Figura 1:** - Fluxograma de localização, identificação e análise preliminar dos documentos. Brasil, setembro de 2022

## Tratamento e análise dos dados

 A extração dos conteúdos (compilação e fichamento de dados) integrou a primeira etapa da análise temática, correspondente à pré-análise, quando se procedeu à leitura preliminar do texto completo para a identificação do contexto, dos autores, dos conceitos-chave e da natureza, lógica e coerência interna dos textos. Nesta etapa, extraíram-se somente as informações relacionadas com o objeto de interesse ^(^^19^^)^ e que respondessem à questão analítica: Quais são as atividades de brincar e interagir recomendadas em guias para crianças com necessidades de saúde especiais? 

 Em seguida, os conteúdos com sentidos similares foram agrupados em unidades de análise em cada texto recuperado, mantendo-se somente os conteúdos aplicáveis à CRIANES-CCC, conforme Figura 2 . 

**Figura 2:** - Procedimento de análise dos documentos recuperados sobre brincar-interagir com CRIANES-CCC 2010-2020. Brasil, setembro de 2022

**Título/País/Ano**	**Conteúdo extraído**	**Recomendação de atividades de brincar-interagir**	**Potencial de aplicação à CRIANES-CCC** ^*^ **- Criança com necessidades de saúde especiais com demandas de cuidados clinicamente complexos**
*Guidelines and Strategies for Children with Special Needs* /Estados Unidos da América/ 2010	Apresenta orientações para que as CRIANES tenham um ambiente acolhedor para brincar e desenvolva suas habilidades e o empoderamento das famílias. Além disso, ilustra para cada condição (crônica, sensorial ou cognitiva), como devem ser implementadas ações e brincadeiras.	Ambiente acolhedor para brincar e ilustra brincadeiras adaptando-as para que crianças, com diferentes condições (crônica, sensorial ou cognitiva) participem.	Colocar um brinquedo mais próximo da criança; Desenvolver ações que estimulam os sentidos (tato, visão e audição); Conversar com a criança sobre o que está sendo feito com ela; Repetir a mesma brincadeira mais de uma vez.
*Care for Child Development. Guide for Clinical Practice* /Recomendação global/2012	Sessões clínicas: brincar e se comunicar com a criança usando atividades e brinquedos apropriados para sua idade e condição (não específica); ser sensível às condições da criança e responder de forma adequada a elas.	Faça perguntas ao cuidador para entender como ele brinca e se comunica com a criança em casa e observe a interação deles.	Ser sensível às condições da criança, respondendo de forma adequada aos seus sinais.	Aproveitar a experiência da criança chamando-a pelo nome ou mostrando um objeto de seu interesse, olhando-a nos olhos. Observar as respostas das crianças e do cuidador às atividades lúdicas e de comunicação.	Usar um objeto colorido (copo) na brincadeira e interação social deixa a criança mais alerta e ativa; Chamar a criança pelo nome; Conversar com ela até obter uma resposta por meio de som ou gestos.
O Direito de Brincar. Um Guia Prático para Criar Oportunidades Lúdicas e Efetivar o Direito de Brincar/Brasil/2013	O material destaca a inclusão e igualdade, para que todas as crianças tenham o direito de brincar. Reforça que os facilitadores da brincadeira devem garantir que crianças com deficiência estejam inseridas na arte, na música, na dança, no teatro e nas histórias.	Todas as crianças devem ter seu direito à brincadeira garantido e estar inseridas na arte, na música e nas histórias.	Os adultos são necessários para serem agentes do brincar nos grupos de crianças.	Tocar e cantar música, movimentar-se e dançar para a criança; Oferecer objetos que a criança consiga pegar (preensão) e que emitam sons (chocalhos); Contar histórias usando livros interativos; Explorar ambientes naturais e contato com plantas, insetos, animais de estimação; Gerenciar programas e espaços de brincadeira próximo ao leito; Focar nas potencialidades e não na falta de habilidade.
Incluir Brincando. Guia do Brincar Inclusivo/Brasil/2012	Seleção de brinquedos, brincadeiras e jogos que permitem a interação e a participação das crianças, independente de suas características.	Selecionar brinquedos, brincadeiras e jogos que facilitam a interação e a participação da criança.	Os brinquedos devem ter cores fortes, pois são estimulantes e ajudam quem tem baixa visão a perceber contrastes, além daqueles que produzam algum som; livros de história, música e fantoches.	Selecionar brinquedos, brincadeiras e jogos facilitadores de interação e participação da criança; Selecionar brinquedos com cores fortes é estimulante, para que crianças com baixa visão percebam os contrastes; Selecionar brinquedos que produzam algum som; Usar histórias publicadas no formato de livros sonoros e musicados; Usar fantoches na contação de histórias.
Diretrizes de estimulação precoce: crianças de 0 a 3 anos com atraso no desenvolvimento neuropsicomotor/Brasil/2016	Elenca brincadeiras e ações interativas, para crianças na faixa etária de 0 a 3 anos, visando a estimulação do desenvolvimento neuromotor de crianças com microcefalia.	Estimular a visão residual, percepção tátil e sonora durante o brincar com outras crianças e cuidadores, apresentando objetos dentro do campo visual de 20 cm, acompanhado de estimulação verbal.	Potencializar a contribuição dos pais ou responsáveis, para que interajam com a criança e estabeleça comunicação e afeto mútuo.	Utilizar brinquedos e objetos grandes adaptados à criança, suas funções sensoriais (auditiva, tátil, proprioceptiva e vestibular), para que olhe e movimente a cabeça (se houver controle de tronco) em direção ao objeto, conversando com ela Usar objetos de tamanhos e tipos variados, coloridos com alto contraste (amarelo e preto e/ou vermelho e branco) brilhante e iluminados; Usar brinquedos sonoros (chocalhos), em seu campo visual e/ou horizontalmente movimentando-os para frente e para trás, de forma lenta; Usar diferentes texturas, para a estimulação tátil, por exemplo: grãos, esponjas, algodão etc.; Disponibilizar móbile, chocalho, brinquedos musicais para morder e que flutuem; Brincar de esconde-esconde;
*Sit Less, Move More, Sleep Well: Active play guidelines for under-fives* /Nova Zelândia/2017	Apontam exemplos de brincadeiras com as crianças acima de cinco anos que são importantes e contribuem para o processo de desenvolvimento. O guia inclui uma abordagem de crianças com deficiência, sem especificar como fazer e o que utilizar nas brincadeiras com esse grupo.	Uso da música; mover objetos coloridos para que a criança possa procurá-los; usar banheira para promover atividades na água; encorajar brincadeiras fora de casa ou parques.	Usar a música como recurso estimulador sonoro; Movimentar objetos coloridos para que a criança o procure; Colocar um pouco de água na banheira para que a criança mantenha contato da pele com a água; Promover atividades na água em condições seguras; Encorajar brincadeiras fora de casa ou parques, sempre que houver oportunidades.
Guia ilustrado para cuidadores de crianças com deficiências neuromotoras/Brasil/2017	Informações sobre cuidados e manejo de crianças com deficiência neuromotora. Apresenta brincadeiras e atividades, recomendando posicionamento correto que promove o desenvolvimento neuromotor.	O banho é uma excelente oportunidade para a interação adulto-criança. Deixe a criança explorar a água e se divertir com ela. Use esponjas com texturas e formas diferentes para passar no corpo da criança; usar brinquedos ou objetos simples para o banho. Peneiras, copinhos e potinhos podem ser usados para brincar e estimular a percepção da criança.	Se possível, carregar a criança olhando para frente para que ela receba maior variedade de estímulos e interaja melhor com o ambiente.	Praças e parques são excelentes para que a criança encontre outras crianças, receba estímulos diferentes daqueles que já tem em casa. Como qualquer outra criança, ela se divertirá tocando plantas e animais.	No banho: Deixar que a criança explore a água e se divirta com ela; Aplicar esponjas com texturas e formas diferentes no corpo; Usar brinquedos ou objetos simples durante o banho (copinhos e potinhos estimulam a percepção da criança). Na mobilidade: Carregar a criança olhando para frente para que receba a maior variedade de estímulos e interaja com o ambiente, sempre que possível; Promover o encontro com outras crianças, em ambientes externos para que receba estímulos diferentes; Estimular que toque plantas e animais ajuda na diversão no e entretenimento.
*South Australian Government. Inclusive play: Guidelines for accessible playspaces* /Austrália/2019	O Guia orienta a criação de espaços que promovam a inclusão e o brincar, criando experiências sensoriais e interativas.	Brincadeiras sensoriais a partir de objetos com diferentes texturas.	Parques com brinquedos inclusivos, com elementos que sejam do alcance da criança e com acesso à cadeira de rodas.	Usar nas brincadeiras objetos com texturas diferentes; Parques com brinquedos inclusivos (balanços com cadeiras de rodas) e que estejam ao alcance das crianças.
*Sensory Design Guidelines: Inclusive Children’s Treatment Centres* /Canadá/2020	O material propõe diretrizes para a concepção de ambientes sensoriais e inclusivos em centros de tratamento infantil, objetivando melhorar as experiências e o bem-estar. Somado a isso, também propõe o desenvolvimento de uma matriz de ambiente sensorial que aborde os cinco sentidos (audição, visão, tato, olfato e paladar).	Criar espaços ao ar livre, tendo contato com elementos da natureza (terra, folhas, água), que contenham objetos com superfícies lisas, ásperas e/ou texturas moles; e utilizar músicas e objetos musicais para promover a interação com as crianças ou músicas ambientes para mantê-las relaxadas,, podem promover interesse, engajamento e aumentar a estimulação tátil.	Criar espaços ao ar livre para estimação sensorial por meio dos elementos da natureza (terra, folhas, água) e superfícies e objetos de diferentes texturas; Utilizar músicas e objetos musicais para promover a interação com as crianças ou músicas ambientes para mantê-las relaxadas.

* CRIANES-CCC = Criança com necessidades de saúde especiais com demandas de cuidados clinicamente complexos

Em um novo movimento analítico, as unidades foram reagrupadas e indexadas em três temas: estimulação sensorial, estimulação à interação adulto-criança, e estimulação das potencialidades.

## Resultados

Dos 200 materiais que resultaram da estratégia de busca, nove atenderam aos critérios de elegibilidade e responderam à questão analítica. Quatro dos nove documentos foram produzidos no Brasil e abordam diretrizes que asseguram o direito de brincar. O brincar inclusivo propõe atividades de estímulo ao desenvolvimento neuromotor de crianças com microcefalia e atraso de desenvolvimento neuromotor, em geral. Dois em nove foram publicados em países da América do Norte (Estados Unidos e Canadá) e recomendam, respectivamente, diretrizes para crianças com deficiência múltipla, e diretrizes sensoriais inclusivas para crianças atendidas em centros de tratamento. Dois guias publicados na Austrália e Nova Zelândia recomendam, respectivamente, o brincar inclusivo e brincadeiras ativas para menores de cinco anos. O guia clínico do Fundo das Nações Unidas para a Infância (UNICEF) recomenda um conjunto de diretrizes para estimular o desenvolvimento da criança com necessidades de saúde especiais.

 As atividades com potencial para promover as potencialidades, a interação e a estimulação sensorial (tato, audição e visão) de CRIANES-CCC foram agrupadas em três temas, conforme Figura 3: estimulação das potencialidades, com um subtema (crença da sociedade no potencial de convivência da criança); estimulação à interação adulto-criança com dois subtemas (voz e expressão corporal do adulto como mediadores do interagir-brincar; gestão da brincadeira); e estimulação sensorial (tato, visão e audição), com quatro subtemas (finalidades dos brinquedos e objetos, posicionamento dos brinquedos e objetos, interagir-brincar no banho, estímulo à mobilidade, sempre que possível). 

**Figura 3:** - Atividades de brincar e interagir recomendadas para crianças com necessidades de saúde especiais com demandas de cuidados clinicamente complexas. Brasil, setembro de 2022

*Estimulação das potencialidades*
*Crença da sociedade no potencial de convivência da criança* encorajamento de brincadeiras fora de casa, em parques ou outras áreas públicas, sempre que houver oportunidades; exposição a brinquedos inclusivos (balanços com cadeiras de rodas) e que estejam ao seu alcance; estímulo para desenvolver brincadeiras na brinquedoteca ou fora do ambiente hospitalar com outras crianças; manutenção do foco nas potencialidades e não na falta de habilidade; promoção do potencial de brincar com atividades na água, em condições de segurança.
*Estimulação à interação adulto-criança*
*Voz e expressão corporal do adulto como mediadores do interagir-brincar* a voz é um objeto do brincar usado para estimular o reconhecimento da pessoa que conversa com a criança; informa sobre o que está sendo feito e facilita a interação; conversando até obter uma resposta (som ou gestos); chamando a criança pelo nome; repetindo a mesma brincadeira mais de uma vez; movimentando o corpo dançando para a criança; brincando de esconde-esconde. *Gestão de brincadeiras* gerenciar programas e espaços de brincadeira próximo ao leito; usar brinquedos, brincadeiras e jogos facilitadores de interação e participação da criança.
*Estimulação sensorial (tato, audição e visão)*
*Finalidades dos brinquedos e objetos:* que produzam som; com cores fortes, para estimular a percepção de contrastes entre aqueles com baixa visão; grandes e adaptados às funções sensoriais (auditiva, tátil, proprioceptiva e vestibular), para estimular o olhar e o movimento da cabeça (se houver controle de tronco) em direção ao objeto; com tamanhos e tipos variados, coloridos com alto contraste (amarelo e preto e/ou vermelho e branco) brilhante e iluminados; com diferentes texturas para promover a estimulação tátil, por exemplo: grãos, esponjas, algodão etc.; que sejam coloridos (copo), usados na brincadeira para estimular a interação deixando a criança mais alerta e ativa; usando a música (para tocar e cantar no ambiente), como recurso sonoro para interagir e relaxar; para morder e que seja flutuante, tais como o móbile, chocalho e objetos musicais; livros sonoros e musicais para usar nas histórias; livros interativos e fantoches, como recursos para a contação de histórias e despertar a atenção da criança; em espaços ao ar livre, contato com a natureza (plantas, terra, folhas, água), contato com insetos e animais de estimação promovem a estimulação sensorial. *Posicionamento dos brinquedos e objetos:* mais próximo (do tato, escuta e visão) da criança; movimentando-os para que a criança os procure; dentro do campo visual e/ou horizontalmente, movimentando brinquedos sonoros (chocalhos) lentamente, para frente e para trás; ao alcance da mão para que consiga pegar (preensão) e produzir sons (chocalhos). *Interagir-brincar no banho:* para explorar a água e se divertir com ela; use no corpo esponjas com texturas e formas diferentes; mantenha contato da pele com a água, colocando pouca água na banheira; use brinquedos ou objetos simples (copinhos e potinhos) que estimulam a percepção da criança. *Estímulo à mobilidade, sempre que possível* transporte a criança mantendo-a posicionada de frente, para que receba a maior variedade de estímulos e interaja com o ambiente; promova o encontro com outras crianças, em ambientes externos para que receba estímulos diferentes.

## Discussão

 Na maioria das vezes, a centralidade dos cuidados está em atender à necessidade de sobrevivência das CRIANES-CCC, priorizando uma assistência de enfermagem que garanta uma respiração pérvia, alimentação segura, pele íntegra e administração de medicamentos de uso contínuo, entre outros aspectos. Esses cuidados de sobrevivência são contínuos, prolongados e intensos, e desviam os profissionais de enfermagem e familiares cuidadores para eles. Nesse sentido, esta seção discute os achados com base nos pressupostos do Cuidado de Collière ^(^^20^^)^ e a concepção de brincar, segundo Walter Benjamin ^(^^21^^)^ . 

 Collière ^(^^20^^)^ compreende os cuidados como aqueles que são vivificantes e convidam à vida, descrevendo-os em sete tipos, porém, sem hierarquizá-los por grau de relevância. O primeiro, de *manutenção* , relaciona-se com as necessidades psicológicas, afetivas e sociais que fazem parte do cotidiano da vida. O segundo, de *reparação* , tem como finalidade a recuperação da doença por meio de um tratamento. O terceiro, de *confortação* , refere-se à renovação e à integração da experiência que promove bem-estar. O de *parecer* corresponde ao quarto, contribui para a valorização da imagem que a pessoa tem de si mesma para fortalecer o senso de identidade e pertencimento ao grupo social em que vive. O de *compensação* , quinto tipo de cuidado, envolve ações substitutivas do que ainda não foi adquirido e/ou compensação por aquilo que a pessoa ainda não é capaz de fazer por si mesma. O de *apaziguamento* , sexto tipo, tem como objetivo o repouso e a liberação de tensões, permitindo apaziguar o que causa turbulência e o que está em desassossego. O sétimo, de *estimulação* visa o estímulo às capacidades fundamentais, dos sentidos, da percepção e da representação; destaca-se uma variedade de cuidados capazes de produzir expectativas, desejos, interesse e afetividade. O brincar, na compreensão de Collière, é um cuidado de manutenção ^(^^20^^)^ . 

Contudo, segundo as características singulares das CRIANES-CCC, o brincar é uma atividade substantiva que mobiliza a vitalidade e o potencial de interação; portanto, aplica-se mais de um tipo de cuidado na perspectiva de Collière.

Por vezes, as condições motoras e psicossociais limitadas dessas crianças impedem que os adultos, profissionais de enfermagem ou familiares cuidadores percebam as potencialidades que elas possuem para participar de interações e brincadeiras que promovem o bem-estar e a conexão com o mundo. A condição limitante e clinicamente complexa se constitui em barreiras ao brincar e interagir não sendo reconhecidos como uma atividade de valor, tanto na comunidade, na escola, como nos serviços de saúde, especialmente no hospital. Nesse sentido, precisa-se lançar um olhar para a unicidade do brincar/brincadeira como atividade humana que conecta a CRIANES-CCC com o mundo de adultos.

 Para uma melhor compreensão do lugar da brincadeira na vida da criança, tomamos o pensamento de Benjamin ^(^^21^^)^ como marco referencial. Segundo o autor, a brincadeira promove libertação, cria para a criança um pequeno mundo que é próprio dela e está cercado pelo mundo de adultos. É desse momento de criação que se extrai o prazer e é ele que faz a criança sentir-se livre. A brincadeira é a faculdade mimética cuja essência é a inovação do “fazer de novo”, de repetir a experiência que a brincadeira proporciona. Ela envolve a capacidade de a criança sair de si, perder-se no outro (podendo ser este outro uma situação, lugar, pessoa, objeto ou palavra) e retornar a si mesma de modo transformado pela experiência de ser outro. Ou seja, no brincar, ela consegue diluir-se no espaço, no lugar e no tempo para dar significado ao objeto que manipula ou aos inúmeros papéis que ele pode representar. 

 Desse modo, as interações são limitadas pela baixa responsividade aos estímulos que são normalmente esperados para as crianças típicas ^(^^22^^-^^23^^)^ . 

 Há uma tendência nos serviços de saúde de valorizar a abordagem biomédica priorizando as questões orgânicas inerentes à condição da criança. No Canadá, os programas institucionais priorizam o “ser criança”, buscando o seu bem-estar, repouso e conforto, centrado no modelo de atenção psicossocial. Por essa razão, investem no potencial de desenvolvimento interacional da criança no seu mundo social ^(^^13^^,^^24^^)^ . 

 Destaca-se que, para as CRIANES-CCC, os cuidados devem estimular suas potencialidades. Contudo, há uma crença ontológica sobre o potencial da criança com paralisia cerebral para interagir durante a brincadeira. Estudo destacou que alguns profissionais não creem no potencial da criança e expressam sua visão ontológica ao dizerem que não há necessidade de retirar do leito para brincar, pois a mesma não fazia nada ^(^^24^^)^ . Criança acamada, com déficit neurológico grave, mesmo tendo brinquedos dispostos em sua cama, com quem nenhuma pessoa interagiu, dando-lhe atenção ou mesmo brincando com ela. A criança esforçava-se para alcançar um dos brinquedos, cansava-se e voltava para observá-los, sem que nenhum adulto utilizasse os objetos como recurso para promover a interação mediada pelo brincar ^(^^24^^)^ . Ambas as situações reforçam a necessidade de se romper preconcepções para que os adultos se engajem em atividades que estimulam as potencialidades das CRIANES-CCC. Essas evidências precisam ser traduzidas nas ferramentas de interação no cuidado vivificante, para que os objetos do brincar e da brincadeira produzam significado para a criança. 

Destaca-se que tais atividades requerem ambiente seguro, sejam aquelas realizadas na ou com água ou outro ambiente que implique em risco à integridade física da criança. É preciso ainda encorajar profissionais de saúde a interagir com a criança, bem como os familiares, para que não deixem essas crianças confinadas às suas casas. Levá-las para ambientes externos à casa, como parques, por exemplo, contribui para prover estímulos mediados por animais e plantas, além de ter a oportunidade de usar brinquedos inclusivos (balanços com cadeiras de rodas) e que estejam ao alcance delas. Contudo, caso haja hospitalização, em especial aquelas prolongadas, a brinquedoteca e ambientes externos ao hospital são também recomendados. Esse conjunto de evidências, quando traduzidos em ferramenta de cuidado, favorece o desenvolvimento do potencial interacional da CRIANES-CCC com o adulto, seja ele profissional de enfermagem ou familiar cuidador, que faz parte do seu mundo social no ambiente do hospital.

 Para Collière, os cuidados implicam em um compromisso pessoal e social do cuidador com aquele que é cuidado ^(^^20^^)^ . O cuidar pode ser somente um gesto simples ou trivial, mesmo assim é um ato de vida. Somado a isso, representa uma diversidade de atividades que visam sustentar e/ou manter a vida, isto é, permitir continuar a viver em todo seu potencial ^(^^25^^)^ . Nesse sentido, criar espaços que estimulem o potencial das CRIANES-CCC é um ato de vida e investimento no seu potencial e não na sua falta de habilidade ^(^^13^^)^ . 

 Na *estimulação das potencialidades* , a sociedade precisa ser acolhedora para a CRIANES-CCC, e as pessoas precisam acreditar no potencial de convivência que elas proporcionam, encorajando-as a brincar fora de casa (parque ou outras áreas públicas), na brinquedoteca hospitalar ou em áreas externas do hospital. Esses ambientes geram oportunidades de encontro com outros, bem como a possibilidade de elas interagirem com outras crianças e brinquedos. Além disso, quando há uma condição segura, deve-se realizar atividades na água, quando incorporado como evidência em ferramentas de cuidado. 

*Estimular as potencialidades* das CRIANES-CCC, mediada por brincadeiras, representa a regulamentação do brincar, um dos direitos fundamentais previstos no Estatuto da Criança e do Adolescente (ECA) (Lei n.º 8.069/1990) ^(^^26^^)^ , para efetivar uma das facetas do cuidado. Além disso, cumpre com outras diretrizes legais previstas no Estatuto da Pessoa com Deficiência (Lei n.º 13.146/2015) ^(^^27^^)^ e na obrigatoriedade de instalar brinquedotecas em unidades de internação pediátrica (Lei n.º 11.104/2005) ^(^^28^^)^ . Somado a isso, a Política Nacional de Atenção Integral à Saúde à Criança (PNAISC) destaca que cabe à família, à comunidade, à sociedade em geral e ao Poder Público a responsabilidade pela efetivação dos direitos da criança, tais como o direito à vida, ao lazer, à convivência familiar e à comunidade, à participação e ao respeito à liberdade ^(^^29^^)^ . As evidências legais, correspondentes às regulamentações na forma de lei e portarias governamentais, fundamentam o direito da CRIANES-CCC ao brincar e precisam ser traduzidas em ferramentas orientadoras de cuidar. 

 Para o grupo de CRIANES-CCC, que é totalmente dependente do outro para brincar e interagir, há necessidade de fomentar iniciativas que *estimulam a interação adulto-criança* . Assim, atividades mediadas pela voz e expressão corporal do adulto tornam-se objeto do brincar, o meio pelo qual se estabelecem a interação e um diálogo capturado pela emissão de sinais ^(^^30^^-^^31^^)^ . É recomendável que, durante a prestação de cuidado, a CRIANES-CCC seja chamada por seu cuidador (profissional ou familiar) pelo seu nome, que ele converse com ela informando sobre o que será feito, brinque de esconde-esconde, e repita essas ações até que se obtenham repostas, como a emissão de sons guturais ou movimentação espontânea ou reflexa. 

 Ao se repetir a mesma brincadeira mais de uma vez, a exemplo do esconde-esconde ou movimentar o próprio corpo (como uma dança) para elas, cria-se um pequeno mundo próprio cercado pelo mundo dos adultos, que proporciona prazer e promove libertação. A *estimulação da interação adulto-criança* , quando se dá de forma repetida, segundo Benjamin significa *mimesis,* ou seja, ocorre o registro das semelhanças e não da igualdade. O “fazer de novo”, envolve a capacidade de criança sair de si, perder-se no outro (podendo ser este outro uma situação, um lugar, uma pessoa, um objeto ou uma palavra) e retornar a si de modo transformado pela experiência de ser outro. Experiências semelhantes permitem que a criança se familiarize com o mundo externo, com ideias, sentimentos e ações, cujo potencial pode ser transformador, permitindo vislumbrar novas possibilidades de relação entre os mundos ^(^^31^^)^ . 

O provimento de experiências semelhantes para as CRIANES-CCC, em especial as que vivem hospitalizações longas, incrementa o repertório relacional que as mantêm conectadas com o mundo e amplia sua convivência social. Portanto, o gerenciamento de programas e espaços de brincadeira, bem como brinquedos e jogos próximo ao leito, que sejam facilitadores da interação e participação da criança, conforme sua habilidade, pode ser enriquecedor, além de prover estímulos sensoriais consistentes e diferentes.

*Para estimular os sentidos (tato, visão e audição)* , é importante conhecer as finalidades de cada brinquedo, em especial aqueles objetos que se aplicam às CRIANES-CCC. Portanto, precisa-se levar em conta que esses materiais devem possuir cores estimulantes, brilhantes, iluminadas, contrastantes, especialmente para aquelas com baixa visão. Além disso, objetos sonoros, de variados tamanhos (bolas grandes) e diferentes texturas estimulam o movimento da cabeça e/ou dos olhos em direção ao objeto, deixando a criança mais alerta e ativa na interação, bem como que sejam adaptados às funções sensoriais que se pretende estimular ^(^^13^^)^ . Além desses brinquedos, como atividades que estimulam os sentidos, recomenda-se cantar ou reproduzir músicas infantis, contar histórias e ler para a criança, assistir TV ^(^^30^^)^ . A brincadeira na interação com CRIANES-CCC precisa ser acompanhada por estímulos sensoriais (audição, visão e tato) para elicitar respostas de prazer que podem ser observadas na expressão facial (sorriso, olhar) ou de outras partes do corpo. O estímulo desperta o interesse pelo som, toque e outros elementos no ambiente, levando a criança a buscá-los, dirigindo seu olhar para a origem da voz e do toque, por exemplo ^(^^31^^-^^32^^)^ . 

 A voz humana é um potente objeto do brincar, seja na conversa, na contação de histórias e livros musicais (sonoros), do mesmo modo que a música (tocar, cantar no ambiente) representa um recurso sonoro de interação e relaxamento. Outras formas de estimular os sentidos é utilizar elementos da natureza (terra, folhas, água) em espaços ao ar livre para que a criança não viva em confinamento imposto pela complexidade clínica. Os dados vão ao encontro de um estudo realizado com crianças com paralisia cerebral que participaram do grupo de contação de histórias multissensoriais em que aconteceram sua participação e interação significativas ^(^^32^^-^^33^^)^ . 

Contudo, para promover a estimulação sensorial, é fundamental que os objetos do brincar sejam disponibilizados horizontalmente dentro do campo sonoro e visual da criança, e que os adultos movimentem brinquedos sonoros lentamente (como o chocalho), para que a criança os procure. Objetos que estimulam a pega devem ser mantidos ao alcance das mãos da criança.

 As sensações experimentadas com a estimulação sensorial (tato, audição e visão) são desencadeadas pelo receptor do nervo neurossensorial ao tomar consciência, reconhecer e interpretar esse estímulo. Ou seja, esse conjunto de experiências sensoriais, seja por meio de música, brinquedos/objetos coloridos e sonoros e texturas diferentes, permite às crianças a percepção de captar e aumentar seu nível de consciência relativo à comunicação e às emoções de prazer ^(^^12^^-^^13^^)^ . 

 Na perspectiva de Collière, o cuidado de estimulação visa despertar as capacidades mais fundamentais, que são o sentir, ouvir e ver, a fim de desenvolver os sentidos e a capacidade motora. Um cuidado estimulador cria expectativas, desejos, interesse e reações afetivas, que são o ponto de partida para a construção do pensamento ^(^^20^^)^ . 

 Consequentemente, despertar respostas de prazer nas CRIANES-CCC é um cuidado de estimulação estratégico quando o cuidador (equipe de enfermagem e/ou familiar) cria um ambiente acolhedor, prazeroso e promotor de bem-estar, o que contribui para a criação de vínculos afetivos baseados nas experiências dessas sensações ^(^^12^^,^^30^^)^ . Para as CRIANES-CCC, os cuidados devem ser transmitidos numa interação com o outro por meio do toque e do contato corpo a corpo, da voz humana, permitindo que elas encontrem seu lugar no mundo e se sintam seguras. Por exemplo, o banho (na banheira ou leito) cria a oportunidade de estimular os sentidos, pois permite que elas explorem a água para se divertir quando a tocam; e usar objetos (esponja) e brinquedos com texturas diferentes que chamem a atenção. 

 Contudo, antes de implementá-las, faz-se necessário saber a condição clínica da criança e a possibilidade para executá-lo dessa forma. Já na mobilidade, caso a criança possa ser transferida do leito para a cadeira de rodas ou carregada no colo, deve-se posicioná-la de modo que a mesma olhe para frente e assim receba mais estímulos e interações vindos de ambientes externos e pessoas com as quais não está habituada ^(^^13^^)^ . 

 Todas essas práticas, quando experimentadas de forma lúdica, suscitam prazer, satisfação e se trocam na reciprocidade de um enriquecimento mútuo ao interagirem e brincarem ^(^^13^^)^ . Portanto, a interação e o brincar são cuidados vivificantes, ou seja, que convidam à vida, restituindo um sentido às crianças que o recebem, em especial, para as CRIANES-CCC que ainda são invisibilizadas quanto ao seu potencial pela sociedade. 

 Para tanto, é necessário repensar o modelo de cuidado vinculado exclusivamente ao paradigma biomédico, para introduzir novas práticas baseadas num paradigma emergente de cuidado centrado nas necessidades de crianças hospitalizadas ^(^^34^^)^ . Assim, faz-se necessário que tanto instituições de saúde quanto os profissionais de saúde se aproximem das produções científicas sobre o brincar e o interagir na mediação do cuidado. O desenvolvimento de atividades interacionais no cuidado pode promover a sociabilidade, despertar o potencial de CRIANES-CCC, reduzir a lacuna entre a assistência e a ciência e fomentar uma prática eficiente e humanizada. Para tanto, guias de cuidados se constituem em ferramentas cujos conteúdos possam instrumentalizar profissionais e familiares sobre atividades estimuladoras. 

## Conclusão

Os guias clínicos recuperados na estratégia de busca apontaram dois grupos de conteúdo. O primeiro trata do brincar e o interagir com destaque para as atividades a serem realizadas com aquelas crianças com necessidades de saúde especiais (CRIANES) (microcefalia, atraso de desenvolvimento neuromotor, baixa visão, deficiências múltiplas). O segundo, indicativos das atividades com potencial de aplicabilidade às CRIANES clinicamente complexas (CRIANES-CCC). Essas evidências, originárias de documentos como fontes primárias de análise, revelam o potencial de tradução e transferabilidade para a produção de ferramenta integradora do interagir no cuidado cotidiano de CRIANES-CCC.

Nessa ferramenta, o brincar e interagir com CRIANES-CCC pode ser agrupado em três conjuntos de atividades: a primeira, são aquelas que estimulam as potencialidades da criança, baseadas na crença ontológica da sociedade e das pessoas na capacidade de convivência no espaço social; a segunda, baseia-se na necessidade de interação adulto-criança como fonte de estimulação com a voz e expressão corporal do adulto e na gestão da brincadeira; a última, reúne atividades que promovem a estimulação sensorial, segundo os tipos e as finalidades de brinquedos e objetos, bem como os cuidados ao posicioná-la durante o banho e a mobilidade.

É importante reforçar, ainda, a necessidade de os profissionais de saúde e familiares romperem com a invisibilidade dessas crianças, investindo e valorizando suas conquistas. O conteúdo específico para a estimulação dos sentidos (tato, visão, audição) de CRIANES-CCC pode ser mediado por objetos do brincar e brincadeiras, desde que o conhecimento seja traduzido em ferramenta orientadora de profissionais de saúde (enfermagem, em especial) e familiares cuidadores, visando à promoção de um cuidado estimulador e vivificante.

 Como limitação do estudo, as guias e diretrizes encontradas não abordam as questões raciais, de gênero e socioeconômicas das crianças com necessidades de saúde especiais. Nesse sentido, há necessidade de realização de pesquisas que possam cobrir a diversidade étnico-racial, de equidade de gênero e social, ampliando o espectro de inclusão social na construção do conhecimento sobre CRIANES-CCC. Além disso, a busca limitou-se aos idiomas inglês e português, com a estratégia de busca aplicada em uma única fonte de informação, o *Google Search **^®^** .*
